# Engineering Natural Product Nanomedicines to Modulate Tumor Immunometabolism for Enhanced Cancer Immunotherapy

**DOI:** 10.3390/molecules31213965

**Published:** 2026-11-03

**Authors:** Yang Xiao, Shaowen Lan, Yuping Dai, Xuenan Liu, Chaowen Huang, Changming Yang, Zhiqi Wang, Linghui Zou

**Affiliations:** 1 School of Pharmacy, University of Southern California, Los Angeles, CA 90033, USA; yxiao473@usc.edu; 2 No. 923 Hospital of Joint Logistic Support Force of PLA, Nanning 530022, China; lsw16666@163.com; 3 School of Pharmacy, Hunan University of Chinese Medicine, Changsha 410208, China; 005060@hnucm.edu.cn (Y.D.); 202630877@stu.hnucm.edu.cn (X.L.); 20262156@stu.hnucm.edu.cn (C.H.); 13911008216@163.com (C.Y.)

**Keywords:** natural products, nanomedicine, tumor immunometabolism, metabolic reprogramming, cancer immunotherapy

## Abstract

Tumor metabolic reprogramming contributes to an immunosuppressive microenvironment and limits the efficacy of cancer immunotherapy. Natural products can regulate multiple metabolic pathways, but their therapeutic application is often restricted by poor bioavailability and insufficient tumor exposure. Nanomedicine offers a means to overcome these limitations while enabling more selective metabolic intervention. This review examines how natural product nanomedicines remodel tumor immunometabolism and influence antitumor immunity. Current evidence shows that these nanomedicines can regulate glucose and lactate metabolism, lipid and amino acid-associated metabolism, and mitochondrial/redox homeostasis. Such metabolic remodeling has been associated with improved CD8^+^ T-cell activity, altered immunosuppressive immune populations, induction of immunogenic cell death, and improved responses to immune checkpoint blockade in selected studies. However, causal links between metabolic regulation and immune remodeling remain insufficiently defined, and metabolic interventions may exert distinct effects across tumor and immune cell populations. Future studies should emphasize cell-specific targeting, direct assessment of metabolic target engagement, and pharmacokinetic-pharmacodynamic relationships. Natural product nanomedicines therefore represent a promising platform for integrating metabolic intervention with cancer immunotherapy.

## 1. Introduction

Cancer immunotherapy has substantially changed the treatment landscape of multiple malignancies, particularly through immune checkpoint blockade (ICB), adoptive cell therapies, and therapeutic vaccination. Durable responses, however, remain restricted to a subset of patients, and many tumors exhibit primary or acquired resistance despite the presence of potentially tumor-reactive immune cells [1,2,3,4]. Increasing attention has therefore shifted from immune checkpoints themselves to the local conditions that determine whether immune cells can remain metabolically competent inside tumors. The tumor microenvironment (TME) is characterized by intense competition for nutrients, accumulation of metabolic by-products, hypoxia, oxidative stress, and spatially heterogeneous access to oxygen and substrates. These conditions are not passive consequences of tumor growth. They actively shape immune-cell differentiation, survival, trafficking, and effector function, creating a close relationship between tumor metabolism and antitumor immunity [5,6,7,8,9]. Tumor immunometabolism has consequently emerged as an important framework for understanding why immune cells that are present within tumors frequently fail to sustain effective antitumor responses [10].

Metabolic competition is particularly relevant because malignant and immune cells rely on many of the same nutrients while using them for different biological purposes. Enhanced glucose consumption and aerobic glycolysis by tumor cells can restrict glucose availability for activated T cells and increase extracellular lactate and acidity, conditions that impair T-cell proliferation and cytokine production while favoring several immunosuppressive populations [11,12,13]. Amino-acid metabolism provides another layer of competition. Glutamine, arginine, tryptophan, and methionine influence both tumor growth and immune-cell function, and their depletion or conversion into immunoregulatory metabolites can suppress T-cell activity or support myeloid and regulatory populations [14,15,16]. Lipid metabolism is similarly context-dependent: altered fatty-acid synthesis, uptake, and oxidation can sustain malignant growth and shape the phenotype of tumor-associated macrophages (TAMs), regulatory T cells (Tregs), and dysfunctional T cells [17,18]. Extracellular adenosine, mitochondrial dysfunction, redox imbalance, and hypoxia further reinforce these suppressive metabolic circuits [19,20,21,22]. Ferroptosis and related redox-dependent forms of cell death have added another dimension to this field. Their induction in tumor cells may increase treatment sensitivity and immunogenicity, whereas excessive lipid peroxidation in immune cells can impair antitumor function, underscoring the importance of cellular context when metabolic pathways are therapeutically manipulated [23,24,25,26].

Natural products are attractive candidates for metabolic intervention because many of them regulate several interconnected pathways rather than acting through a single molecular target. Polyphenols, quinones, terpenoids, flavonoids, and other natural compounds have been reported to influence glycolysis, mitochondrial respiration, redox homeostasis, lipid metabolism, autophagy, inflammatory signaling, and regulated cell death [27,28,29]. Compounds such as curcumin, resveratrol, shikonin, dihydroartemisinin, celastrol, honokiol, and chlorogenic acid are particularly relevant because their pharmacological effects extend across both malignant and immune compartments. This multitarget activity is potentially useful in a metabolically interconnected TME, where inhibition of a single enzyme may be insufficient because tumors can compensate through alternative substrates or pathways. At the same time, pleiotropic activity complicates mechanistic interpretation. A change in immune infiltration after natural-product treatment does not necessarily indicate direct immune regulation, and a change in tumor metabolism does not by itself establish that the resulting immune response is metabolically driven. Defining the sequence from metabolic target engagement to immune-cell remodeling is therefore essential if natural products are to be developed as rational immunometabolic therapeutics rather than broadly acting anticancer agents. Recent literature has increasingly recognized both the metabolic potential of phytochemicals and the need to distinguish direct metabolic regulation from secondary effects caused by cytotoxicity, stress responses, or tumor regression [30]. Among these compounds, shikonin and resveratrol have been investigated for glycolytic regulation, while dihydroartemisinin and celastrol are frequently associated with redox regulation, ferroptosis, and mitochondrial dysfunction. Curcumin has also been explored for its effects on glycolytic and mitochondrial pathways. These diverse activities provide a pharmacological basis for developing natural product-based immunometabolic nanomedicines, although their therapeutic relevance requires careful mechanistic validation.

A second limitation is pharmaceutical rather than mechanistic. Many bioactive natural compounds have poor aqueous solubility, limited chemical stability, rapid metabolism, short systemic exposure, and insufficient accumulation in tumors. These properties can prevent the concentrations required for metabolic modulation from being reached at the relevant tumor or immune-cell population [31,32]. Nanomedicine offers several ways to address this problem [33]. Liposomes, polymeric nanoparticles, metal-organic or metal-phenolic systems, biomimetic vesicles, carrier-free nanoparticles, hydrogels, and stimulus-responsive formulations can improve solubility and pharmacokinetics, protect unstable compounds, control release, and alter tissue or cellular distribution [34,35]. More importantly for immunometabolic therapy, nanoscale delivery can potentially separate desirable and undesirable metabolic effects. Tumor-cell glycolysis or redox buffering may be suppressed while limiting exposure of metabolically vulnerable lymphocytes; TAM- or stromal-targeted delivery may remodel suppressive populations without imposing the same metabolic stress on effector T cells. Nanotechnology is therefore not merely a strategy to improve the bioavailability of natural products. It provides a means of controlling where, when, and in which cell population metabolic intervention occurs, a feature that is particularly important because the same metabolic pathway can support antitumor immunity in one cell type while promoting immune suppression in another. Recent developments in tumor-responsive nanomaterials and immunometabolism-oriented nanomedicine increasingly reflect this shift from passive drug delivery toward cell-selective modulation of the TME [36,37].

Against this background, this review focuses specifically on how natural product nanomedicines can remodel tumor-associated metabolism and how these metabolic changes influence antitumor immunity and immunotherapy. A literature search was performed using PubMed, Web of Science, and Scopus databases, covering studies published mainly from 2015 to 2026. Relevant studies were identified using combinations of keywords related to natural products, nanomedicine, tumor metabolism, immunometabolism, and cancer immunotherapy. Studies were included when they investigated natural product-based nanomedicine systems with experimentally evaluated metabolic regulation and/or immune-related outcomes. In this review, particular attention was given to studies with direct metabolic measurements, including glucose uptake, lactate production, ATP generation, mitochondrial respiration, GSH/GPX4 regulation, lipid peroxidation, and metabolomic profiling. Importantly, metabolic alterations in tumor cells were distinguished from direct metabolic regulation of immune cells, and studies were interpreted according to whether metabolic changes were mechanistically linked to immune-cell functional alterations or only accompanied by immune responses. To further distinguish the mechanisms and supporting evidence, the studies discussed in this review are considered within four categories: (i) direct metabolic regulation of immune cells; (ii) tumor-cell metabolic alterations associated with indirect immune benefits; (iii) metabolic stress or regulated cell death followed by immune activation; and (iv) immunomodulatory effects without direct evidence of metabolic involvement. For multicomponent formulations, mechanistic and immunological outcomes are attributed to an individual natural product only when supported by component-specific controls; otherwise, they are interpreted as effects of the complete formulation.

Rather than broadly cataloguing natural compounds or nanocarrier types, we organize the available evidence around three major metabolic domains: glucose and lactate metabolism, lipid and amino-acid metabolism, and mitochondrial/redox homeostasis. We then examine whether these interventions are associated with improved T-cell function, altered immunosuppressive populations, and enhanced responses to cancer immunotherapy. Particular attention is given to the strength of the evidence linking metabolic alteration to immune outcome, because many existing studies report these events in parallel without demonstrating causality. Finally, we discuss cell-specific targeting, pharmacokinetic-pharmacodynamic relationships, formulation reproducibility, safety, and clinical translation. This framework is intended to distinguish natural-product delivery that merely produces antitumor effects from nanomedicine strategies that genuinely use metabolic reprogramming to reshape the tumor immune microenvironment and enhance cancer immunotherapy.

## 2. Tumor Immunometabolism as a Therapeutic Target

### 2.1. Glucose and Lactate Metabolism

Metabolic reprogramming is a fundamental feature of cancer biology. Among various metabolic alterations, enhanced glycolysis is one of the most frequently observed changes in tumors. Many cancer cells preferentially convert glucose into lactate even under oxygen-rich conditions, providing intermediates for biosynthesis and supporting rapid proliferation. However, increased glycolytic activity also reshapes the metabolic landscape of the TME. Nutrient availability, metabolite accumulation, and metabolic competition collectively influence the function of infiltrating immune cells [38,39].

Glucose is essential for the activation and expansion of effector T cells. Following antigen stimulation, CD8^+^ T cells increase glucose uptake and glycolytic activity to support proliferation, cytokine production, and cytotoxic responses. In contrast, highly glycolytic tumor cells consume substantial amounts of glucose, creating a nutrient-restricted environment that limits immune-cell metabolism. Chang et al. first demonstrated that metabolic competition between tumor cells and T cells represents an important mechanism of immune suppression in tumors [40]. Increased glucose consumption by cancer cells reduced glucose availability for infiltrating T cells, resulting in impaired glycolysis and decreased IFN-γ production. Restoring glucose availability through metabolic intervention improved T-cell function and enhanced tumor control.

The influence of glucose metabolism on immunity is not limited to energy production. Glycolytic intermediates can directly regulate immune-cell signaling pathways. Ho et al. identified phosphoenolpyruvate (PEP) as a metabolic checkpoint controlling antitumor T-cell responses [41]. Under glucose-limited conditions, reduced PEP levels impaired Ca^2+^-NFAT signaling and weakened T-cell effector function, whereas increasing PEP production enhanced cytokine production and antitumor activity. These findings indicate that tumor-associated glucose depletion can affect immune responses through both metabolic insufficiency and altered intracellular signaling.

Enhanced glycolysis also results in excessive lactate production and accumulation within the TME. Lactate was previously regarded mainly as a metabolic end product, but it is now recognized as an important regulator of tumor-immune communication. Elevated lactate concentrations negatively affect cytotoxic lymphocytes by suppressing T-cell and NK-cell activity. Further, lactate dehydrogenase A (LDHA)-associated lactate production impairs tumor immune surveillance by reducing IFN-γ production and limiting the function of antitumor lymphocytes [42]. In addition to directly affecting lymphocytes, lactate contributes to the establishment of an immunosuppressive myeloid compartment. Colegio et al. provided early evidence that tumor-derived lactate promotes macrophage polarization toward a tumor-supportive phenotype, accompanied by increased expression of arginase 1 (ARG1) and VEGF [43]. Later studies further revealed that lactate sensing through GPR132 participates in tumor-macrophage communication and promotes breast cancer progression [44]. Together, these findings suggest that lactate acts as a signaling metabolite capable of reshaping immune-cell phenotypes within tumors.

The impact of glucose depletion and lactate accumulation varies among different immune populations. While these metabolic conditions restrict the activity of conventional effector T cells, Tregs display considerable metabolic adaptability. Foxp3-driven metabolic remodeling allows Tregs to reduce their dependence on glycolysis and maintain oxidative metabolism, enabling their survival in glucose-poor environments [45]. Tumor-infiltrating Tregs can further utilize lactate as an alternative metabolic substrate. Watson et al. demonstrated that lactate supports the metabolic fitness and suppressive function of intratumoral Tregs through MCT1-mediated uptake [46]. This metabolic advantage may contribute to the preferential enrichment of Tregs in highly glycolytic tumors and further strengthen local immune suppression.

The relationship between tumor glycolysis and immune checkpoint therapy has attracted increasing attention. Beyond affecting immune-cell abundance and function, lactate metabolism may influence the outcome of immunotherapy. Kumagai et al. reported that lactate uptake through MCT1 promoted NFAT1 activation and increased PD-1 expression in Tregs within highly glycolytic tumor environments [47]. This metabolic adaptation affected the balance between effector T-cell activation and regulatory suppression during PD-1 blockade therapy. These observations provide a link between tumor metabolic characteristics and variability in immune checkpoint inhibitor responses.

Overall, glucose and lactate metabolism represent a key metabolic interface between tumor cells and immune populations within the TME. Increased glucose consumption by tumor cells limits the metabolic capacity of effector lymphocytes, whereas lactate accumulation reshapes immune-cell composition and function by suppressing cytotoxic responses and supporting immunosuppressive populations. These metabolic alterations contribute to the formation of an immune-suppressive environment and reduced sensitivity to immunotherapy. However, the complexity of tumor immunometabolism should be considered when developing metabolic interventions. Many metabolic pathways are shared by malignant cells and immune cells, and systemic metabolic inhibition may cause undesirable effects on normal immune function. A better understanding of cell-specific metabolic dependencies and the regulatory mechanisms underlying immune-cell adaptation will be essential for developing more precise strategies to remodel the tumor immune microenvironment.

### 2.2. Lipid and Amino Acid Metabolism

Beyond glucose utilization, lipid and amino acid metabolism represent another major layer of metabolic regulation within the TME. Tumor cells require large amounts of lipids and amino acids to support membrane synthesis, energy production, redox balance, and rapid proliferation. Meanwhile, immune cells rely on the same nutrients to maintain activation, differentiation, and effector functions. The competition for these metabolic resources, together with the accumulation of abnormal metabolites, contributes to immune dysfunction and tumor immune escape [48,49].

Recent studies have highlighted lipid metabolism as an important determinant of CD8^+^ T-cell fate in tumors. Lipids are essential components of cellular membranes and serve as substrates for energy metabolism; however, excessive lipid exposure within tumors can generate metabolic stress and impair immune-cell function. Cholesterol accumulation represents one of the best-characterized examples. Increased cholesterol levels in the TME induce endoplasmic reticulum stress and activate XBP1 signaling in CD8^+^ T cells, accompanied by increased expression of exhaustion-associated molecules such as PD-1, TIM-3, and LAG-3 [50]. This cholesterol-driven metabolic stress promotes a dysfunctional T-cell state and limits antitumor immunity.

The relationship between lipid metabolism and CD8^+^ T-cell dysfunction has attracted increasing attention in recent years. A 2024 review by Tang et al. summarized emerging evidence that lipid uptake, synthesis, oxidation, and storage collectively regulate CD8^+^ T-cell responses during tumor progression [51]. Excessive lipid accumulation is frequently observed in tumor-infiltrating lymphocytes and is associated with impaired mitochondrial function, altered signaling, and reduced effector activity. These findings suggest that lipid metabolism is not simply a consequence of immune dysfunction but an important factor shaping T-cell differentiation and exhaustion.

Specific lipid transport pathways also influence immunosuppressive cell populations. FATP2-mediated lipid uptake has been identified as an important metabolic feature of tumor-associated neutrophils and MDSCs. Veglia et al. demonstrated that FATP2 promotes arachidonic acid uptake and prostaglandin E2 production, thereby enhancing the suppressive activity of MDSCs [52]. Similarly, CD36-mediated uptake of oxidized lipids contributes to lipid peroxidation and ferroptotic stress in tumor-infiltrating CD8^+^ T cells. Blocking CD36 or reducing lipid peroxidation restored T-cell effector function and improved responses to PD-1 blockade [53]. Recent studies further indicate that ferroptosis represents a complex metabolic interface between lipid metabolism and immunity, as lipid peroxidation can either promote tumor-cell death or impair immune-cell viability depending on the cellular context [54].

Amino acid metabolism provides another critical mechanism through which tumors regulate immune responses. Unlike glucose metabolism, amino acid regulation often involves selective depletion of essential nutrients or accumulation of immunosuppressive metabolites. Tumor cells and immunosuppressive myeloid cells actively reshape amino acid availability, creating metabolic conditions unfavorable for effective T-cell responses.

Arginine metabolism is a classic example of amino acid-mediated immune suppression. Tumor-associated macrophages and MDSCs frequently express high levels of ARG1, resulting in local arginine depletion. Because activated T cells require extracellular arginine for proliferation and T-cell receptor expression, ARG1-mediated arginine consumption leads to impaired lymphocyte activation and reduced antitumor responses [55]. In addition to arginine, tryptophan metabolism has attracted considerable attention because of the immunosuppressive activity of kynurenine metabolites generated through indoleamine 2,3-dioxygenase 1 (IDO1) and tryptophan 2,3-dioxygenase (TDO) pathways. Tryptophan depletion restricts T-cell proliferation, whereas kynurenine accumulation activates AhR signaling and promotes regulatory immune programs [56].

Recent advances have expanded the understanding of amino acid metabolism from individual pathways to integrated metabolic networks. Wetzel et al. discussed the interaction between arginine, glutamine, and branched-chain amino acid metabolism within the TME, emphasizing that amino acid utilization by tumor cells and immune cells is highly interconnected rather than isolated [57]. Glutamine metabolism is particularly important because it supports nucleotide synthesis, mitochondrial activity, and redox regulation. Tumors with strong glutamine dependence can restrict glutamine availability for immune cells, whereas metabolic differences between tumor cells and lymphocytes may provide opportunities for selective intervention. Leone et al. demonstrated that glutamine blockade suppressed tumor metabolism while promoting adaptive metabolic changes in T cells, resulting in improved antitumor immunity [58].

The importance of amino acid metabolic regulation in cancer immunotherapy has continued to increase. Recent reviews have summarized several amino acid-metabolizing enzymes, including GLS1, ARG1, IDO1, TDO, and IL4I1, as potential targets for overcoming metabolic immune suppression [16]. These studies also highlight a major challenge: amino acid metabolism is required for both tumor survival and normal immune-cell function, making selective targeting essential.

Collectively, lipid and amino acid metabolism contribute to tumor immune escape through multiple mechanisms, including abnormal lipid accumulation, oxidative lipid stress, nutrient competition, and immunosuppressive metabolite production. Lipid metabolic remodeling is closely linked with CD8^+^ T-cell exhaustion and ferroptotic vulnerability, whereas amino acid metabolic pathways regulate immune-cell activation and differentiation. Understanding these metabolic dependencies provides a foundation for developing precise strategies to reshape the tumor immune microenvironment and improve therapeutic responses.

### 2.3. Mitochondrial Metabolism and Redox Homeostasis

Mitochondria represent a central metabolic hub that integrates energy production, biosynthetic activity, and redox regulation in immune cells. Although activated T cells initially increase glycolysis to support rapid expansion, mitochondrial oxidative metabolism remains essential for maintaining long-term functional capacity, particularly in memory and stem-like T-cell populations [59]. Within the TME, persistent antigen stimulation, hypoxia, nutrient limitation, and oxidative stress continuously challenge mitochondrial homeostasis, resulting in impaired metabolic flexibility and progressive immune dysfunction [60].

Mitochondrial impairment has been recognized as an early metabolic feature of exhausted T cells. Unlike transient metabolic changes during physiological immune activation, tumor-infiltrating lymphocytes frequently exhibit reduced mitochondrial mass, impaired oxidative phosphorylation (OXPHOS), and decreased respiratory capacity. Scharping et al. demonstrated that the TME suppresses PGC-1α-dependent mitochondrial biogenesis in infiltrating T cells, leading to metabolic insufficiency and reduced antitumor activity [61]. This work established mitochondrial biogenesis as an important determinant of T-cell fitness in tumors.

The connection between mitochondrial dysfunction and T-cell exhaustion was further clarified through studies examining immune checkpoint signaling. PD-1 signaling alters T-cell metabolic programming by reducing glucose utilization, mitochondrial activity, and metabolic adaptability. Bengsch et al. showed that bioenergetic defects occur during the early stage of CD8^+^ T-cell exhaustion and precede terminal functional loss [62]. These findings suggest that metabolic dysfunction contributes to exhaustion development rather than simply reflecting an exhausted state.

Mitochondrial quality control and dynamics also influence the fate of tumor-reactive T cells. Mitochondrial fusion, fission, and biogenesis regulate the balance between effector differentiation and long-term persistence. Buck et al. demonstrated that mitochondrial dynamics control T-cell fate through metabolic programming, with enhanced mitochondrial fusion associated with memory-like functional states [63]. Recent studies have suggested that mitochondrial integrity and metabolic fitness are important determinants of CD8^+^ T-cell survival and functional persistence during antitumor responses.

The importance of mitochondrial fitness in cancer immunotherapy has been increasingly highlighted by recent advances. In 2024, Simula et al. demonstrated that mitochondrial metabolism supports CD8^+^ T-cell migration into solid tumors. Using three-dimensional tumor models, the study showed that T-cell motility depends strongly on mitochondrial glucose and glutamine oxidation, and that enhancing mitochondrial activity improves intratumoral infiltration and CAR-T-cell antitumor efficacy [64]. This finding expands the role of mitochondrial metabolism beyond energy supply, suggesting that mitochondrial function also determines whether immune cells can effectively access tumor regions.

Mitochondrial dysfunction can also be transmitted between cells within the TME. Recent work published in Nature revealed that tumor cells can transfer mitochondria carrying mitochondrial DNA alterations to tumor-infiltrating lymphocytes. These transferred mitochondria induce metabolic abnormalities, impair memory formation, and reduce T-cell effector function, providing a previously unrecognized mechanism of immune escape [65]. In contrast, mitochondrial transfer can also be therapeutically exploited. A 2024 Cell study demonstrated that stromal-cell-derived mitochondrial transfer through intercellular nanotubes enhanced CD8^+^ T-cell mitochondrial mass, respiratory capacity, tumor infiltration, and resistance to exhaustion, resulting in improved antitumor activity [66]. These studies indicate that mitochondrial regulation within the TME is dynamic and bidirectional, involving both pathological mitochondrial exchange and potential therapeutic mitochondrial restoration.

Hypoxia and oxidative stress represent additional drivers of mitochondrial dysfunction in tumors. Persistent antigen stimulation under hypoxic conditions increases mitochondrial stress and reactive oxygen species (ROS) accumulation. Scharping et al. further demonstrated that continuous stimulation combined with hypoxia rapidly induces mitochondrial stress and accelerates CD8^+^ T-cell exhaustion through impaired mitochondrial adaptation [67]. Excessive ROS disrupts mitochondrial integrity, alters metabolic signaling, and promotes exhaustion-associated transcriptional programs. Recent studies have also shown that cytokine-mediated regulation of mitochondrial metabolism can directly influence T-cell bioenergetic capacity. For example, Meteorin-like (METRNL), a metabolically active cytokine within tumors, was found to disrupt mitochondrial function in CD8^+^ T cells by altering E2F-PPARδ signaling, resulting in reduced oxidative phosphorylation and impaired antitumor responses [68].

Beyond exhaustion, mitochondrial metabolism also affects the therapeutic responsiveness of immune cells. Mitochondrial respiratory capacity, including spare respiratory capacity, has been implicated in CD8^+^ T-cell memory development and persistence. Moreover, mitochondrial metabolic programming has been shown to influence the differentiation and functional properties of adoptively transferred T cells [69,70]. These findings highlight the importance of metabolic fitness for maintaining durable antitumor immune responses.

**Figure 1:**
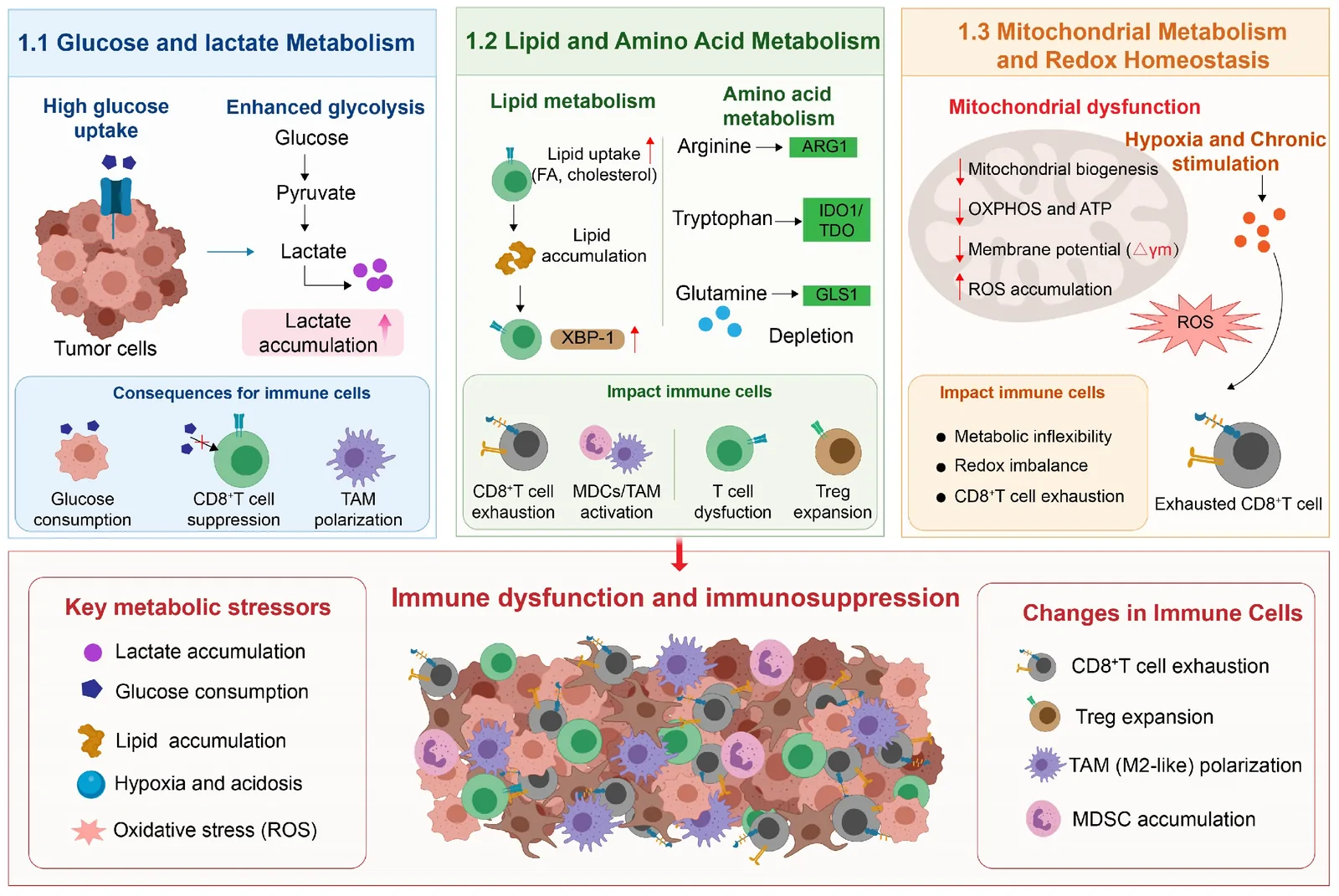
Metabolic dysregulation in the tumor microenvironment and its impact on antitumor immunity. The three numbered panels represent (**1.1**) glucose and lactate metabolism, (**1.2**) lipid and amino acid metabolism, and (**1.3**) mitochondrial metabolism and redox homeostasis. The schematic illustrates how altered nutrient availability, lactate accumulation, and mitochondrial and redox stress may affect antitumor and immunosuppressive immune-cell populations. Arrows indicate the proposed direction of metabolic or immunological effects, while ↑ and ↓ indicate increases and decreases, respectively. TME, tumor microenvironment; TAMs, tumor-associated macrophages; MDSCs, myeloid-derived suppressor cells; Tregs, regulatory T cells. The symbols and colors used in the schematic are explained in the corresponding legends within the figure.

Overall, mitochondrial metabolism and redox homeostasis represent the final metabolic layer connecting environmental stress with immune-cell dysfunction. Glucose, lipid, and amino acid availability determine the metabolic pressure experienced by immune cells, whereas mitochondrial function determines whether these cells can adapt and maintain antitumor activity. Understanding mitochondrial vulnerabilities within immune cells provides an important foundation for developing strategies aimed at restoring immune competence and improving cancer immunotherapy. Collectively, these metabolic alterations shape the functional state of both antitumor and immunosuppressive immune populations, thereby contributing to an immunosuppressive TME (Figure 1).

## 3. Natural Product Nanomedicines for Metabolic Remodeling

### 3.1. Targeting Glucose and Lactate Metabolism

Glucose metabolism is an attractive target for natural product nanomedicines because many solid tumors maintain high glycolytic activity even under oxygen-replete conditions. This metabolic preference supports rapid ATP production and biosynthesis, but it also changes the extracellular environment. Glucose becomes less available to infiltrating lymphocytes, while lactate accumulates and contributes to local acidosis and immune suppression. As discussed above, these changes are particularly unfavorable for cytotoxic T-cell activity [71]. The challenge is to suppress tumor glycolysis without imposing the same metabolic restriction on antitumor immune cells [48].

Direct evidence in this area is still relatively limited. Many natural compounds are known to regulate glycolysis in their free form, yet only a small number of nanoformulations have experimentally demonstrated changes in glucose uptake, glycolytic enzymes, ATP production, or lactate levels. Shikonin is one of the better-supported examples. It inhibits pyruvate kinase M2 (PKM2), a glycolytic enzyme frequently upregulated in malignant cells. A shikonin-loaded, hyaluronic acid-modified mesoporous polydopamine nanosystem (SHK@HA-MPDA) was developed for colorectal cancer liver metastasis and showed clear inhibition of PKM2-dependent glycolysis. Treatment reduced both lactate and ATP production in colorectal cancer cells, while tumor lactate levels were also decreased in vivo [72].

The biological consequences extended beyond energy restriction. Lower lactate availability was accompanied by reduced recruitment of myeloid-derived suppressor cells (MDSCs), increased NK and CD8^+^ T-cell populations, and higher granzyme B and IFN-γ levels in tumors, suggesting an association between glycolytic inhibition and immune microenvironment remodeling. Dendritic-cell activation was also enhanced. Exogenous lactate partially reversed several of these effects, strengthening the link between glycolytic inhibition, lactate reduction, and remodeling of the immune microenvironment [72]. The same study connected lactate metabolism with epithelial-mesenchymal transition (EMT), showing that reduced lactate flux interfered with PKM2-associated signaling involved in metastatic progression.

More recent nanoplatforms have used this metabolic vulnerability as part of combination treatment rather than relying on glycolytic inhibition alone. Shikonin-loaded hollow Fe-metal-organic framework (MOF) nanoparticles, for example, were designed to combine metabolic intervention with microwave thermal therapy. Shikonin and ROS generated by the Fe-MOF system suppressed tumor-cell glycolysis, reducing the energy supply needed to tolerate thermal stress. The metabolic intervention consequently increased tumor sensitivity to microwave treatment in vitro and in vivo [73]. Although immune remodeling was not the main endpoint of this study, it illustrates a useful engineering principle: glycolytic inhibition can be incorporated into a nanoplatform to weaken metabolic adaptation to another therapeutic stress.

Resveratrol provides a second, mechanistically distinct example. Early work using resveratrol-loaded PEG-PLA nanoparticles demonstrated suppression of glucose metabolism in CT26 colorectal cancer. 18F-FDG uptake was reduced both in cultured tumor cells and in tumors following intravenous administration, providing direct evidence that the nanoformulation retained the metabolic activity of resveratrol in vivo [74]. Tumor growth was delayed, and survival improved compared with empty nanoparticle controls. This study did not examine immune-cell metabolism, but it was among the earlier demonstrations that nano-delivered natural products could alter tumor glucose utilization rather than merely increase cytotoxic drug exposure.

A more direct connection between glucose metabolism and antitumor immunity was later demonstrated using a dual-responsive polyplex for the co-delivery of resveratrol and PD-L1 siRNA. Instead of simply shutting down glucose utilization, the formulation was designed to rebalance tumor-cell glycolysis and mitochondrial OXPHOS. Resveratrol suppressed glycolytic metabolism and promoted a shift toward mitochondrial respiration, while PD-L1 silencing simultaneously removed an immune checkpoint signal [75]. This metabolic change reduced glucose consumption and lactate production in tumor cells, leaving a metabolically less restrictive microenvironment for immune cells.

The immune effects were consistent with this metabolic remodeling. Treatment increased intratumoral CD8^+^ and CD4^+^ T-cell infiltration and IFN-γ production, while reducing Tregs and MDSCs [75]. The study is particularly relevant to immunometabolic nanomedicine because the metabolic component was not treated as an independent cytotoxic mechanism. Instead, tumor glucose metabolism was deliberately manipulated to support immune activation and improve the response to PD-L1 inhibition.

These studies also reveal an important distinction between glycolysis inhibition and immunometabolic remodeling. Complete glucose deprivation is unlikely to be desirable because activated CD8^+^ T cells themselves depend on glucose and glycolytic flux for proliferation and effector function. A more useful strategy is to preferentially reduce excessive glucose consumption by tumor cells and limit lactate production. This may relieve metabolic competition without directly suppressing immune-cell metabolism. Nanocarriers can contribute through tumor-selective accumulation, stimulus-responsive release, or delivery to cells with high expression of relevant metabolic targets.

The current evidence remains concentrated around a small number of natural compounds, particularly shikonin and resveratrol. Direct targeting of lactate transport is even less developed. MCT1 and MCT4 regulate lactate exchange across the plasma membrane and represent attractive nodes for future natural product nanomedicines, yet convincing studies combining a natural compound, a defined nanoplatform, direct MCT/lactate measurements, and immune outcomes remain scarce. Similar gaps exist for GLUT1, HK2, and LDHA. This limited evidence should not be interpreted as a weakness of the concept; rather, it defines an area where mechanistically designed nanomedicines could provide clearer links between drug delivery, metabolic remodeling, and antitumor immunity.

### 3.2. Targeting Lipid and Amino Acid Metabolism

Lipid metabolism offers a different therapeutic entry point from glucose metabolism. In this setting, current natural product nanomedicines have been developed less frequently to suppress fatty-acid synthesis or cholesterol metabolism directly. The available evidence is instead concentrated around lipid redox homeostasis and ferroptosis. This distinction is important. Ferroptosis is driven by iron-dependent accumulation of phospholipid peroxides and is closely controlled by cellular antioxidant systems, particularly the cystine-glutathione (GSH)- glutathione peroxidase 4 (GPX4) axis [76]. Natural compounds capable of disturbing this balance can therefore be coupled with iron-containing or redox-responsive nanomaterials to amplify lipid oxidative stress selectively within tumors [77]. Accordingly, this section focuses primarily on lipid redox regulation and ferroptosis, for which more direct experimental evidence is available, while other lipid and amino acid metabolic pathways are discussed as emerging directions with comparatively limited evidence.

Dihydroartemisinin (DHA) is currently one of the clearest examples. Its endoperoxide bridge can react with intracellular iron to generate reactive intermediates, but rapid clearance and insufficient iron availability often limit this effect in vivo. Han et al. addressed both problems by co-delivering a cholesterol derivative of DHA and pyropheophorbide-iron in ZnP-based core-shell nanoparticles. The formulation stabilized DHA, increased tumor exposure, and provided an exogenous iron source to enhance ferroptotic stress [78]. The resulting tumor-cell death was reported to be associated with immunogenic effects. In colorectal tumor models, this treatment was associated with increased tumor immunogenicity and improved antitumor efficacy when combined with PD-L1 blockade.

Subsequent systems have placed greater emphasis on the GSH-GPX4 defense machinery. MOF-based nanoplatforms have also been investigated for ferroptosis-oriented therapy through the regulation of tumor-associated oxidative stress [79]. Fe-MnO_2_ nanosheets loaded with DHA were designed to combine iron-dependent radical generation with disruption of antioxidant protection. The formulation consumed redox buffering capacity, inhibited GPX4 activity, and increased lipid peroxide accumulation, providing evidence of ferroptosis-associated lipid oxidative stress rather than merely increased total ROS production [80]. These changes were accompanied by immunogenic cell death, greater CD4^+^ and CD8^+^ T-cell infiltration, and altered macrophage polarization in hepatocellular carcinoma models.

The same principle can be extended from tumor cells to selected immunosuppressive populations. Fe-based MOFs have attracted interest as drug-delivery platforms because their metal-containing structures can be incorporated into redox-responsive therapeutic designs [81]. A DHA-loaded iron MOF nanoreactor, DHA@MIL-101, accumulated efficiently in tumor-associated macrophages and induced ferroptosis-associated DNA damage [82]. This was followed by activation of the cGAS-STING-IRF3 pathway and a shift toward an M1-like phenotype. Ferroptosis inhibition weakened both STING activation and macrophage reprogramming, supporting a mechanistic connection between the metabolic perturbation and the immune phenotype. A related biomimetic system exploited acidic tumor conditions to release DHA together with iron, while conversion of Fe^3+^ to Fe^2+^ consumed intratumoral GSH. The resulting oxidative pressure induced ferroptosis in both tumor cells and M2-type macrophages and alleviated immunosuppression [83].

Metal-phenolic networks based on Fe(III)/Fe(II) coordination have been explored for biomedical applications, providing a materials-based rationale for constructing iron-containing natural product nanomedicines [84]. Shikonin-based nanomedicines provide an example of integrating redox modulation with cancer immunotherapy. A shikonin-Fe(III) supramolecular nanocomplex incorporated into tragacanth gum increased lipid oxidative damage and was associated with ferroptosis-related and immunogenic cell death responses in bladder tumors. Combination with PD-1 blockade produced stronger antitumor effects than either treatment alone. However, the improved combination efficacy should not, by itself, be interpreted as proof of pharmacological synergy or checkpoint-blockade sensitization [85]. More recently, an Fe-shikonin nanomedicine developed for head and neck squamous cell carcinoma showed GSH-responsive iron release, increased lipid peroxidation, and reduced GPX4 expression. Ferroptotic stress promoted dendritic-cell maturation and enhanced cytotoxic CD8^+^ T-cell responses, with the immune changes further supported by single-cell transcriptomic analysis [86].

Not every ferroptosis-oriented formulation has demonstrated immune remodeling. A cross-linked lipoic-acid nanodrug carrying DHA, for example, enhanced DHA solubility and produced GPX4-dependent ferroptosis in endometrial carcinoma models, but immune endpoints were not central to the study [87]. Celastrol-loaded ZIF-8 nanoparticles have likewise been developed to exploit ferroptosis-associated vulnerability. Such studies remain useful evidence that nanoengineering can amplify lipid-redox disruption, but they should be distinguished from systems in which immune consequences have been directly measured [88].

These findings also raise a more complex issue for immunometabolic therapy. Ferroptosis is not intrinsically beneficial simply because it kills malignant cells. Excessive lipid peroxidation in tumor-infiltrating CD8^+^ T cells can compromise their effector function, as discussed in Section 2.2. The therapeutic objective is therefore not maximal ferroptosis throughout the TME, but cell-selective control of ferroptotic stress. Current DHA nanoreactors illustrate this principle: iron availability, carrier uptake, tumor acidity, GSH abundance, and cellular targeting can be engineered to determine where the oxidative reaction occurs. The same strategy may eventually be used to induce ferroptosis preferentially in tumor cells or selected suppressive myeloid populations while protecting cytotoxic lymphocytes.

Evidence for direct regulation of other lipid pathways by natural product nanomedicines is much thinner. Fatty-acid synthesis, fatty-acid oxidation, cholesterol handling and lipid uptake are all important regulators of tumor immunity, yet convincing nanoformulations that combine a defined natural compound with direct measurements of these metabolic processes and subsequent immune responses remain scarce. The current literature is therefore much more accurately described as lipid-redox remodeling than as broad lipid metabolic reprogramming.

A similar limitation applies to amino acid metabolism. Natural products have individually been reported to affect glutamine utilization, arginine metabolism and the tryptophan-kynurenine pathway, but these activities cannot automatically be attributed to their nanoformulations. Under the stricter criterion of requiring a natural product nanomedicine together with direct metabolic measurements, convincing studies targeting glutaminolysis, ARG1-dependent arginine depletion, or IDO/TDO-driven tryptophan metabolism remain limited. One related nanoengineering approach has combined curcumin with the IDO-pathway inhibitor indoximod to relieve immunosuppression, but because the amino-acid metabolic intervention is provided primarily by indoximod rather than curcumin itself, it does not constitute direct evidence that nano-delivered curcumin remodels tryptophan metabolism.

The cystine-cysteine-GSH axis currently provides the clearest bridge between amino acid metabolism and natural product nanomedicine. Cysteine availability supports GSH synthesis, which in turn maintains GPX4-dependent detoxification of lipid hydroperoxides. Several DHA- and shikonin-based nanosystems exploit this vulnerability through GSH consumption or GPX4 suppression, effectively coupling amino-acid-derived antioxidant metabolism to lipid peroxidation and ferroptosis. This convergence may explain why the evidence base is presently much stronger for ferroptotic redox remodeling than for classical glutamine, arginine, or tryptophan pathways [76,79,83,86].

The imbalance in the literature also points to an opportunity. Future systems could move beyond ferroptosis and directly interrogate glutamine consumption, kynurenine production, arginine availability, or cell-specific amino-acid transport in tumors. Combining these measurements with immune-cell phenotyping would help determine whether natural product nanomedicines can regulate amino acid competition with the same mechanistic clarity already emerging for glucose-lactate and lipid-redox metabolism.

### 3.3. Targeting Mitochondrial and Redox Metabolism

Mitochondria provide a particularly attractive target for natural product nanomedicines because they integrate energy production, redox balance, calcium signaling, and cell-death control. Cancer cells often retain considerable mitochondrial flexibility even when glycolysis is dominant, allowing them to adjust OXPHOS and antioxidant defenses under therapeutic stress. Natural compounds can interfere with these processes, but intracellular exposure alone does not guarantee efficient mitochondrial engagement. Nanoengineering offers an additional level of control by directing the compound toward mitochondria or triggering its release in response to organelle-specific conditions [89,90].

Celastrol provides one of the clearest examples. This quinone methide triterpene can disturb mitochondrial function, but its poor solubility, short circulation time, and systemic toxicity restrict effective exposure in vivo. Geng et al. conjugated celastrol to a poly(N-oxide)-based polymer that self-assembled into nanoparticles and displayed intrinsic mitochondrial targeting [91]. The formulation accumulated in tumor-cell mitochondria and disrupted mitochondrial membrane integrity more efficiently than free celastrol. The biological response was not limited to mitochondrial injury. Immunogenic cell death was enhanced, and tumor-cell PD-L1 expression was reduced, producing stronger antitumor activity in vivo.

More explicit evidence for metabolic interference came from a mitochondria-targeted celastrol delivery system developed for breast cancer. The nanoparticles contained a TPP-modified core and a tumor-responsive outer layer, allowing sequential tumor-cell uptake, lysosomal escape, mitochondrial localization, and celastrol release in the relatively alkaline mitochondrial environment [92]. Mitochondrial respiration was directly evaluated by oxygen consumption rate, together with mitochondrial membrane potential. Celastrol delivery impaired respiratory function and caused pronounced mitochondrial damage in 4T1 cells. The formulation also produced substantial tumor inhibition in vivo. This study is useful because it moves beyond the common use of ROS as a surrogate endpoint and directly interrogates mitochondrial bioenergetic function.

Mitochondrial redox stress can also be amplified through calcium dysregulation. Zheng et al. incorporated curcumin into acid-sensitive PEGylated CaCO_3_ nanoparticles, generating a calcium nanomodulator in which curcumin acted as a Ca^2+^ enhancer [93]. Following tumor-associated activation, the system induced mitochondrial Ca^2+^ overload. Ultrasound further amplified this response, producing increased ROS and stronger mitochondrial injury. The resulting cell death was immunogenic, and treatment suppressed both tumor growth and metastatic progression. Here, curcumin was not simply delivered as a conventional cytotoxic agent. Its biological activity was integrated with the inorganic carrier to perturb mitochondrial ion and redox homeostasis.

Other natural compounds have been incorporated into organelle-targeted carriers using a similar rationale. A recent triptolide nanoliposome was modified with folic acid for tumor targeting and triphenylphosphonium for mitochondrial localization. In Huh-7 hepatocellular carcinoma cells, the dual-targeted formulation showed greater mitochondrial accumulation, mitochondrial fragmentation, loss of membrane potential, and increased intracellular ROS than non-targeted triptolide liposomes [94]. Tumor inhibition reached approximately 79% in the xenograft model, while systemic toxicity was reduced. Immune remodeling was not examined, so this study is better interpreted as evidence for nano-enabled mitochondrial/redox targeting rather than immunometabolic reprogramming.

The connection between mitochondrial damage and immunity becomes clearer when mitochondrial components themselves function as danger signals. An ATP-responsive metal-polyphenol network reported in 2026 was engineered to deliver celastrol directly to tumor-cell mitochondria [95]. Following mitochondrial accumulation and ATP-triggered drug release, celastrol caused membrane depolarization and leakage of mitochondrial DNA into the cytoplasm. Cytosolic mtDNA subsequently activated the cGAS-STING pathway, coupling mitochondrial injury to innate immune sensing. The treatment promoted immunogenic cell death, antigen presentation, and cytotoxic T-cell infiltration, and further improved the response to PD-1 blockade in postoperative tumor models.

This finding illustrates an important feature of mitochondrial immunometabolism. Mitochondrial disruption does not influence immunity only through ATP depletion or ROS production. Release of mtDNA and other mitochondrial danger signals can convert organelle stress into innate immune activation. In this context, mitochondrial targeting provides a potential route for natural products to simultaneously influence tumor metabolism and immune signaling within the same therapeutic process.

A related strategy has recently been explored in triple-negative breast cancer. A mitochondria-targeted biomimetic nanoplatform combined celastrol-calcium complexes with chloroquine to interfere with the adaptive response to mitochondrial injury. Celastrol-induced Ca^2+^ overload and ROS accumulation damaged mitochondria, whereas chloroquine prevented compensatory mitophagy and lipophagy from clearing damaged organelles and lipid deposits [96]. Persistent mitochondrial-ER stress increased DAMP release and promoted CD8^+^ T-cell-mediated antitumor immunity. The study highlights a different engineering concept: mitochondrial stress can be strengthened not only by increasing its initial magnitude, but also by preventing tumor cells from repairing the metabolic damage.

These studies also show why ROS should be interpreted carefully in natural product nanomedicine. Increased total intracellular ROS alone does not demonstrate mitochondrial metabolic reprogramming. Stronger evidence comes from measurements of oxygen consumption, respiratory-chain activity, mitochondrial membrane potential, mitochondrial ROS, ATP production, redox buffering, mitochondrial calcium, or organelle-specific damage. The distinction is relevant because many nanoformulations of curcumin, resveratrol, quercetin, and related compounds report elevated ROS and apoptosis without demonstrating that mitochondria are the primary metabolic target. Such studies provide useful pharmacological information but offer weaker support for mitochondrial immunometabolism.

The same caution applies to immune effects. Mitochondrial damage in tumor cells can promote immunogenic cell death (ICD), mtDNA sensing and antitumor immunity, whereas mitochondrial dysfunction in CD8^+^ T cells is generally detrimental to their persistence and effector capacity. Therapeutic benefit therefore depends on where mitochondrial stress is induced. Tumor-selective accumulation, organelle targeting and stimulus-responsive release become especially valuable in this setting. They allow mitochondrial metabolism to be disrupted in malignant cells while limiting exposure of metabolically vulnerable lymphocytes.

**Figure 2:**
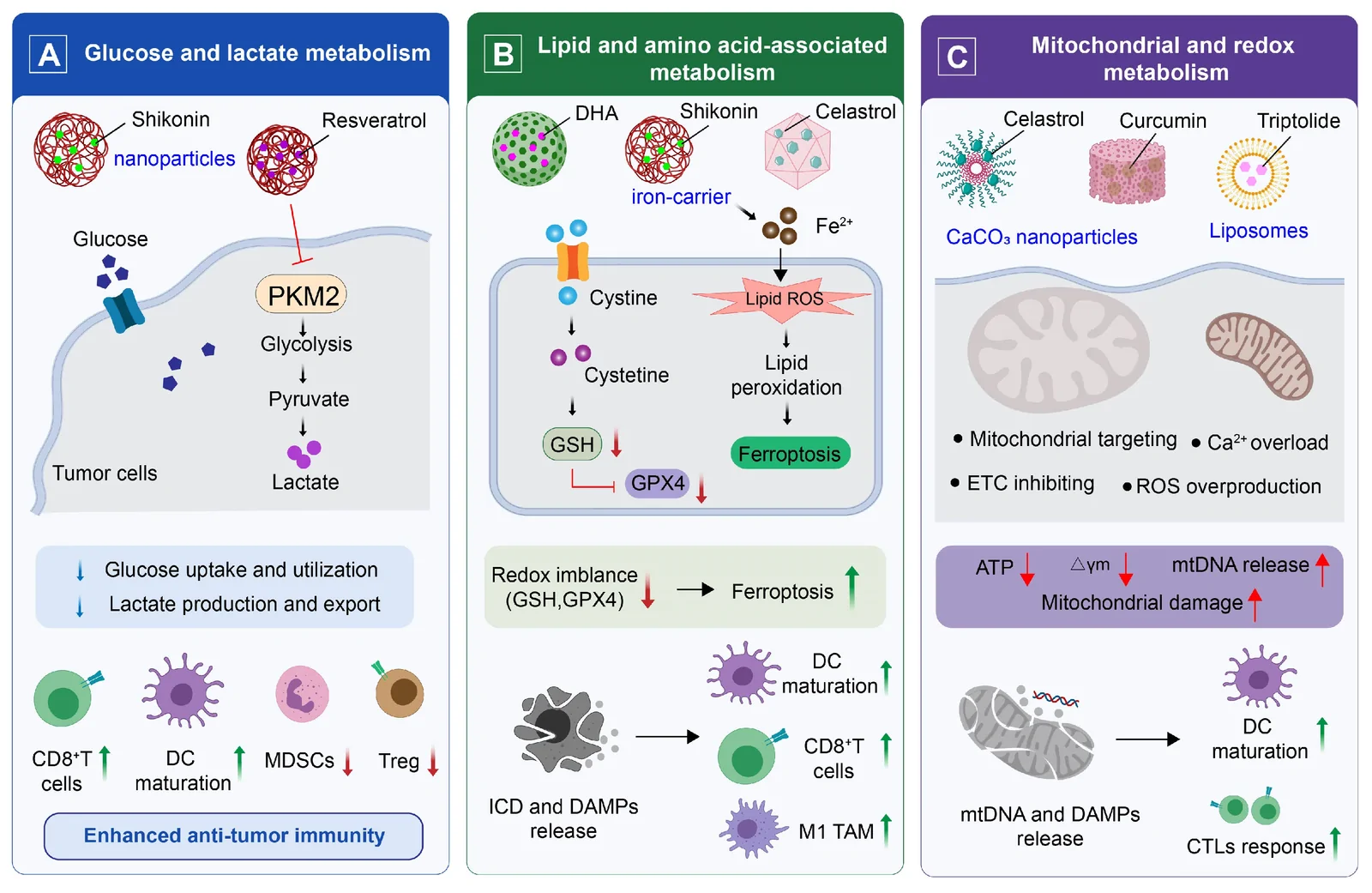
Natural product nanomedicines for metabolic remodeling of the tumor immune microenvironment. The schematic summarizes representative strategies targeting glucose/lactate metabolism, lipid redox homeostasis, and mitochondrial function. Arrows indicate proposed regulatory relationships, while ↑ and ↓ denote increases and decreases in the indicated metabolic processes or markers. ROS, reactive oxygen species; GSH, glutathione; GPX4, glutathione peroxidase 4; OXPHOS, oxidative phosphorylation.

Current evidence remains more developed for inducing mitochondrial damage in tumor cells than for directly restoring mitochondrial fitness in immune cells. Few natural product nanomedicines have been designed specifically to enhance CD8^+^ T-cell OXPHOS, mitochondrial biogenesis, spare respiratory capacity, or redox resilience. This represents an important gap. Future systems could move beyond the current paradigm of “mitochondria-targeted tumor killing” toward cell-selective metabolic engineering, combining suppression of tumor mitochondrial adaptation with preservation-or active restoration-of mitochondrial fitness in antitumor lymphocytes. Together, these studies show that nanoformulation can extend the metabolic actions of natural products across glucose/lactate metabolism, lipid and amino acid-associated metabolism, and mitochondrial/redox homeostasis, as illustrated in Figure 2. Representative natural product nanomedicines and their major metabolic targets and effects are summarized in Table 1. Overall, the strength of evidence varies across the metabolic pathways discussed in this section. Glucose/lactate metabolism and ferroptosis-associated redox regulation are supported by relatively more direct experimental measurements, whereas evidence for the modulation of glutamine, arginine, and tryptophan metabolism by natural product nanomedicines remains limited. Importantly, direct metabolic changes and their causal contributions to immune responses have not been consistently demonstrated, highlighting the need to distinguish relatively well-supported mechanisms from emerging therapeutic directions.

## 4. Immunometabolic Reprogramming to Enhance Cancer Immunotherapy

### 4.1. Restoration of CD8^+^ T-Cell Effector Function

CD8^+^ T cells are highly sensitive to metabolic conditions within the tumor microenvironment. Persistent glucose competition, lactate accumulation, oxidative stress, and mitochondrial dysfunction can limit their proliferation and effector activity even when tumor antigens are recognized [97]. Natural product nanomedicines may relieve these constraints in two ways: by reducing the metabolic pressure imposed by tumor cells or by acting more directly on T-cell metabolic fitness. Current evidence is stronger for the first mechanism, although recent studies have begun to explore direct metabolic regulation of tumor-infiltrating T cells [98]. Importantly, increased CD8^+^ T-cell infiltration should be distinguished from improved effector function, reversal of exhaustion, memory formation, or restored metabolic fitness. These outcomes require separate functional and metabolic assessments rather than changes in cell abundance alone.

The shikonin-based nanoplatform discussed in Section 3.1 also provides evidence of immune changes associated with lactate reduction in colorectal cancer liver metastasis [72]. Treatment increased intratumoral CD8^+^ T-cell infiltration and IFN-γ and granzyme B levels while reducing MDSC accumulation. Importantly, exogenous lactate partially reversed several of these effects, supporting a contribution of lactate remodeling to the observed immune response. This study provides a relatively direct example of how reducing tumor-derived metabolic pressure can restore local cytotoxic immunity.

A similar principle was demonstrated with resveratrol, although the metabolic intervention involved a broader shift in cellular energy metabolism. Dual-responsive polyplexes carrying resveratrol and PD-L1 siRNA suppressed tumor glycolysis, reduced glucose consumption and lactate production, and increased reliance on mitochondrial oxidative phosphorylation [75]. The remodeled metabolic environment contained more CD8^+^ and CD4^+^ T cells and fewer Tregs and MDSCs, together with increased IFN-γ production. In this case, metabolic intervention was combined with checkpoint silencing, allowing nutrient availability and inhibitory signaling to be addressed simultaneously. The resulting immune response was stronger than that achieved by either component alone [75].

Recent work with curcumin has provided a more specific link between tumor glycolysis and dysfunctional T-cell states. NGR-modified curcumin nanovesicles were developed for triple-negative breast cancer and suppressed tumor glycolytic activity [99]. Rather than assessing T-cell infiltration alone, the study examined CD8^+^ T-cell senescence and implicated TLR9/mTOR signaling in the response. Curcumin nanodelivery reduced the senescent phenotype of CD8^+^ T cells and was associated with improved antitumor activity and a better response to immune checkpoint therapy [99]. This finding expands the consequences of tumor metabolic competition beyond acute loss of effector activity. Sustained metabolic stress may also push tumor-reactive lymphocytes toward persistent dysfunctional states.

Direct manipulation of T-cell metabolism remains much less common, but emerging evidence suggests that it is feasible. CD93-targeted PLGA nanoparticles carrying resveratrol were recently designed to regulate the mitochondrial metabolism of CD8^+^ tumor-infiltrating lymphocytes [100]. The formulation promoted AIF-associated mitochondrial regulation and enhanced oxidative phosphorylation in CD8^+^ T cells. Improved mitochondrial metabolism was accompanied by stronger T-cell effector activity and better tumor control, particularly when combined with PD-1 blockade [100]. Unlike approaches that first alter tumor-cell metabolism and then indirectly benefit lymphocytes, this strategy treats T-cell metabolic fitness itself as a therapeutic target.

Lipid-redox remodeling provides another route to T-cell activation, although the relationship is less direct. Dihydroartemisinin-containing Fe-MnO_2_ nanosheets disrupted the GSH-GPX4 antioxidant system and increased lipid peroxidation in tumor cells [80]. The resulting ferroptotic stress promoted immunogenic cell death and was accompanied by greater CD4^+^ and CD8^+^ T-cell infiltration. Similar immune consequences have been reported for Fe-shikonin nanomedicines. In these systems, depletion of GSH, increased lipid peroxidation, and suppression of GPX4/SLC7A11 enhanced ferroptotic tumor-cell death, followed by dendritic-cell maturation and increased CD8^+^ T-cell responses [86,101]. The T cells are therefore not necessarily metabolically reprogrammed directly. Instead, metabolic vulnerability in tumor cells is converted into antigenic and inflammatory signals that improve T-cell priming and recruitment.

This distinction is important when interpreting ferroptosis-based immunometabolic therapy. Lipid peroxidation is beneficial when it is preferentially amplified in malignant cells, but excessive lipid oxidative stress in CD8^+^ T cells can impair their survival and cytotoxic function. A successful nanomedicine should therefore create metabolic asymmetry within the TME: ferroptotic pressure should be high in tumor cells while antioxidant and mitochondrial fitness are preserved in antitumor lymphocytes. Tumor-targeted iron-polyphenol systems and stimuli-responsive carriers offer one route toward such spatial control.

Other forms of redox-regulated cell death may operate through a similar mechanism. Copper-chlorogenic acid nanoparticles depleted intracellular GSH and induced cuproptotic and pyroptotic stress in tumor cells [102,103]. The subsequent release of damage-associated signals promoted dendritic-cell maturation and CD8^+^ T-cell activation. Again, the immune effect arose primarily from metabolically induced tumor-cell stress rather than direct modification of T-cell metabolism. Such systems nevertheless show how metabolic cell death can be translated into adaptive immunity when the nanoplatform produces sufficient immunogenic signaling.

Mitochondrial regulation may offer a broader opportunity for restoring durable T-cell responses. Mitochondrial fitness determines ATP production, redox buffering, persistence, and memory formation in activated lymphocytes. Most natural product nanomedicines developed to date have focused on damaging tumor-cell mitochondria rather than supporting immune-cell mitochondria. Some of these systems still enhance T-cell immunity indirectly. Curcumin-loaded calcium nanomodulators, for example, induce mitochondrial Ca^2+^ overload and oxidative stress in tumor cells, resulting in immunogenic cell death and subsequent antitumor immune activation [93]. Mitochondrial damage in the malignant compartment can therefore serve as an upstream trigger for adaptive immunity, even when T-cell bioenergetics are not directly targeted.

An emerging alternative is to correct pathological mitochondrial signaling rather than simply increase mitochondrial injury. Honokiol-loaded nanomicelles have been reported to regulate SIRT3-associated mitochondrial homeostasis and reduce aberrant mitochondrial stress signaling in hepatocellular carcinoma [104]. The remodeled microenvironment showed increased cytotoxic T-cell and mature dendritic-cell populations together with reduced myeloid-derived suppressor cells (MDSCs). Although this mechanism differs from direct metabolic enhancement of T cells, it illustrates how controlling mitochondrial homeostasis in the tumor compartment may indirectly preserve a more permissive immune environment.

Taken together, current studies suggest two routes for restoring T-cell function with natural product nanomedicines. The more established approach targets tumor metabolism-reducing glucose and lactate pressure or inducing immunogenic metabolic stress-to improve T-cell access, priming, and effector activity. A newer strategy acts directly on the metabolic state of CD8^+^ T cells, particularly their mitochondrial fitness and oxidative phosphorylation. Evidence for the latter remains limited, but it may represent the more precise form of immunometabolic intervention. Future studies should move beyond T-cell abundance and routinely examine metabolic parameters alongside exhaustion, senescence, cytokine production, cytotoxicity, and memory formation. This would clarify whether increased T-cell infiltration reflects genuine metabolic restoration or simply accompanies tumor regression.

### 4.2. Reprogramming of Immunosuppressive Cells

TAMs, MDSCs, and Tregs adapt readily to the metabolic conditions of the tumor microenvironment. Among these populations, TAMs currently provide the clearest evidence for metabolic intervention by natural product nanomedicines. The available studies point to two main routes. Some formulations alter tumor-derived metabolites, particularly lactate, and thereby create conditions less favorable for an M2-like phenotype. Others impose ferroptotic or redox stress directly on macrophages, leading to their repolarization or selective depletion [105,106]. However, the M1/M2 classification represents a simplified description of macrophage phenotypes and does not fully capture TAM heterogeneity. Changes in a limited number of polarization markers should therefore be interpreted as phenotypic shifts rather than definitive evidence of functional macrophage reprogramming. Similarly, reductions in total MDSC abundance do not distinguish monocytic from granulocytic subsets, while increased DC maturation markers do not necessarily demonstrate functional antigen cross-presentation.

Lactate remodeling provides a relatively direct link between tumor metabolism and macrophage phenotype. Shikonin-loaded nanomedicines that suppress PKM2-dependent glycolysis reduce lactate accumulation and are accompanied by a less suppressive myeloid environment [72]. More direct evidence was obtained with emodin-loaded hollow Fe_3_O_4_ nanoparticles coated with galactose-modified erythrocyte membranes [107]. The nanoparticles preferentially interacted with M2-like TAMs while emodin suppressed LDH-associated lactate production. Reduced lactate levels were accompanied by increased CD16/32 expression and TNF-α release, consistent with a shift toward an M1-like macrophage phenotype [107]. This study is particularly relevant because lactate reduction and TAM repolarization were incorporated into the same nanotherapeutic design rather than being inferred from separate experiments.

An earlier shikonin-based system reached a similar endpoint through simultaneous targeting of tumor cells and macrophages. Mannosylated lactoferrin nanoparticles were used to co-deliver shikonin and JQ1 to cells expressing mannose receptor or LRP-1 [108]. Shikonin suppressed tumor glucose metabolism, while the formulation promoted TAM repolarization. DC maturation and CD8^+^ T-cell infiltration increased, whereas Treg abundance declined. JQ1 provided an additional PD-L1 inhibitory component [108]. The study is useful despite its earlier publication date because it directly connects glucose metabolic suppression with remodeling of several immune compartments within the same experimental system.

Redox and ferroptotic stress offer another route to macrophage remodeling. DHA-loaded MIL-101 nanoreactors induced ferroptosis-associated damage in TAMs and activated the cGAS-STING-IRF3 pathway [82]. Macrophages subsequently shifted toward an M1-like state. Ferroptosis inhibition attenuated STING activation and macrophage reprogramming, providing stronger mechanistic support than studies based solely on changes in M1/M2 markers. A related DHA-based biomimetic nanoinducer consumed GSH during Fe^3+^-to-Fe^2+^ conversion and amplified iron-dependent oxidative stress in both tumor cells and M2-like TAMs [83]. In this case, the suppressive macrophage population was targeted through ferroptotic vulnerability rather than conventional repolarization.

Baicalein-based carrier-free nanoparticles provide a more recent example of redox remodeling coupled to macrophage regulation. Baicalein and chlorin e6 were assembled into BC nanoparticles, in which baicalein reduced intratumoral GSH and enhanced photodynamic therapy-induced ferroptosis [109]. At the same time, treatment promoted M1 macrophage polarization and TNF-α production. These effects cooperated with immunogenic cell death to reshape the suppressive microenvironment [109]. The metabolic and macrophage responses were demonstrated within the same study, although the available evidence does not establish that GSH depletion itself directly drives macrophage polarization.

Shikonin can also be integrated directly into iron-dependent redox nanomedicines. Fe(III)-shikonin metal-phenolic networks disassemble in the tumor microenvironment to release Fe^2+^ and shikonin [110]. The resulting oxidative stress induced ferroptosis and necroptosis, accompanied by immunogenic cell death. Tumor-derived antigens and inflammatory signals subsequently promoted DC maturation and antigen cross-presentation, macrophage repolarization, and cytotoxic T-cell infiltration [110]. This represents a broader form of immune remodeling in which metabolic cell death in malignant cells initiates changes in both innate and adaptive immune compartments.

Celastrol-based systems extend this concept beyond iron-dependent ferroptosis. A metal-coordinated carrier-free hydrogel containing celastrol, glycyrrhizic acid, and Cu^2+^ generated strong oxidative and copper-associated stress within tumors [111]. Treatment increased M1-like macrophages, enhanced DC activation, and promoted T-cell infiltration. These changes also improved the response to PD-L1 blockade. The macrophage effect should be interpreted as part of a broader remodeling process rather than evidence that celastrol directly rewires macrophage metabolism, but the study shows how redox-active natural products and metal ions can be combined to simultaneously perturb tumor metabolism and relieve immune suppression.

MDSCs appear to be influenced mainly through changes in the surrounding metabolic environment. Reduction of tumor glycolysis and lactate production by shikonin- or resveratrol-containing nanomedicines has been accompanied by lower MDSC accumulation [72,75]. Honokiol-loaded nanomicelles provide another example [104]. Regulation of SIRT3-associated mitochondrial homeostasis and senescence signaling in tumor cells was associated with fewer MDSCs, together with increases in mature dendritic cells (DCs) and cytotoxic T cells [104]. These findings support a connection between tumor metabolic state and MDSC accumulation, but they do not yet demonstrate direct metabolic reprogramming within the MDSCs themselves.

Evidence for Tregs is similarly indirect. Resveratrol-mediated remodeling of glycolysis and OXPHOS reduced the intratumoral Treg population [75], while shikonin-containing iron nanoplatforms have also been associated with lower Treg abundance after ferroptosis and ICD induction [86,101]. The shikonin/JQ1 system likewise suppressed Tregs while reducing tumor glucose metabolism [108]. These findings are consistent with a less immunosuppressive metabolic environment, but changes in Treg number should not be equated with direct Treg metabolic reprogramming. Measurements of Treg glycolysis, fatty-acid oxidation, mitochondrial respiration, or amino-acid utilization remain uncommon in this literature.

Taken together, the evidence is considerably stronger for TAMs than for MDSCs or Tregs. TAMs can be affected through lactate reduction, redox remodeling, ferroptotic stress, or selective metabolic vulnerability, and several studies now link these interventions to macrophage repolarization within the same experimental system. For MDSCs and Tregs, most observations remain downstream consequences of broader tumor metabolic remodeling. This difference is worth preserving rather than grouping all suppressive immune cells under the same mechanism.

Cell specificity will also become increasingly important. Ferroptosis, ROS accumulation, and mitochondrial stress may be useful when concentrated in tumor cells or suppressive macrophages, but the same disturbances can compromise DCs or cytotoxic lymphocytes. Nanoengineering offers a way to control this distribution through macrophage-targeting ligands, biomimetic membranes, metal-polyphenol assembly, and microenvironment-responsive release. Future studies should combine these delivery strategies with direct metabolic profiling of isolated TAMs, MDSCs, and Tregs. Measurements of lactate utilization, glycolytic flux, oxidative phosphorylation, lipid handling, and amino-acid metabolism would help distinguish true metabolic reprogramming from secondary changes in immune-cell abundance.

### 4.3. Implications for Cancer Immunotherapy

The therapeutic value of immunometabolic remodeling is ultimately determined by whether it improves the response to established immunotherapies. This point is particularly important for ICB, which can restore tumor-reactive T-cell activity but often fails when the surrounding microenvironment remains metabolically hostile or strongly immunosuppressive [112]. Natural product nanomedicines are increasingly being used to address both problems. Rather than acting as standalone cytotoxic agents, some systems reduce metabolic competition, induce immunogenic metabolic stress, or remodel suppressive stromal and immune compartments before or during PD-1/PD-L1 blockade [113].

One of the clearest examples involves glycolytic reprogramming. In hepatocellular carcinoma treated with incomplete radiofrequency ablation, residual tumors showed increased PKM2 expression together with enhanced immunosuppression and PD-L1 expression. Wang et al. designed a liposomal nanomedicine containing shikonin, anti-PD-L1, and spermidine to counteract this adaptive response [114]. Shikonin inhibited PKM2-dependent glycolysis, while the antibody component blocked PD-L1 and spermidine supported CD8^+^ T-cell function. Combined with radiofrequency ablation, the formulation promoted dendritic-cell maturation, relieved local immunosuppression, and suppressed both residual tumor growth and lung metastasis. This study provides a direct example of metabolic intervention being incorporated into an ICB-oriented therapeutic design rather than used as an independent antitumor mechanism.

A related shikonin nanoplatform was developed for head and neck squamous cell carcinoma, where resistance to ICB is frequently associated with an immune-excluded phenotype. CGKRK-modified shikonin lipid nanoparticles induced necroptotic tumor-cell death and disrupted mismatch-repair signaling, which activated a cGAS-dependent interferon response and increased PD-L1 expression [115]. Addition of anti-PD-1 increased dendritic-cell and CD8^+^ T-cell infiltration, and incorporation of Mn^2+^ further strengthened cGAS-STING activation and cytotoxic T-cell differentiation. Although the dominant mechanism in this study was not glycolytic remodeling, these findings suggest that natural product nanomedicines may enhance antitumor responses when combined with checkpoint blockade.

Ferroptotic and cuproptotic stress may also contribute to improved antitumor efficacy in combination with ICB. The DHA-containing ferroptosis systems discussed in Section 3.2 already show that iron-dependent lipid peroxidation can increase tumor immunogenicity and improve PD-L1 blockade [78]. More recently, a DHA nano-hydrogel was designed to enhance copper-dependent cell death in triple-negative breast cancer [116]. The formulation increased copper-associated cell-death responses and altered tumor redox status, accompanied by improved antitumor efficacy when combined with anti-PD-1 therapy in vivo. These findings support the therapeutic potential of combining metabolic cell-death induction with checkpoint blockade, although the contribution of each mechanism to the improved immune response should be interpreted according to the available validation experiments.

Celastrol-based nanomedicines provide another example of combining metabolic stress with cancer immunotherapy. Cel-Cu nanoparticles use celastrol as both a natural anticancer compound and a copper ionophore. At the same time, celastrol scavenges intracellular GSH, thereby removing an important barrier to cuproptosis. The formulation induced self-amplified copper-associated metabolic stress, accompanied by ICD-related responses and enhanced antitumor immunity [117]. The findings support a link between GSH depletion, copper-dependent cell-death processes, and immune activation, while the extent to which each step causally contributes to the downstream immune effects depends on the corresponding mechanistic validation. When combined with ICB, metastatic tumors were effectively controlled in a lung metastasis model. These findings suggest an association between copper accumulation, GSH depletion, immune activation, and improved antitumor efficacy in combination with checkpoint blockade.

The interaction between lipid-rich stromal compartments and ICB has also attracted attention. Cancer-associated adipocytes can promote immune escape through free fatty acid and adipokine signaling and can increase both membrane PD-L1 and PD-L1-containing extracellular vesicles in triple-negative breast cancer [118]. Celastrol-loaded liposomes were recently used to disrupt this adipocyte-associated immunosuppressive environment [119]. Treatment reduced PD-L1 expression and PD-L1^+^ extracellular vesicle release, altered suppressive immune populations, and improved the response to anti-PD-L1 therapy in orthotopic 4T1 tumors. Although this study does not provide the same depth of metabolic flux data as the shikonin or DHA systems, it broadens the concept of immunometabolic targeting by showing that stromal nutrient and adipokine signals can influence checkpoint resistance.

Mitochondrial and redox stress can also be exploited to increase tumor immunogenicity before checkpoint therapy. A photothermal-responsive Cu_9_S_8_ nanoplatform carrying curcumin produced ROS, mitochondrial dysfunction, and immunogenic cell death following NIR irradiation [120]. Curcumin simultaneously downregulated PD-L1 through inhibition of EGFR-STAT3 signaling. Combination with anti-PD-1 increased cytotoxic T-cell infiltration and dendritic-cell maturation while reducing Tregs, leading to stronger tumor suppression than either modality alone. This formulation illustrates how metabolic organelle damage, immune-checkpoint regulation, and spatially controlled drug release can be combined within one platform.

Curcumin has also been incorporated into nanomedicines that directly integrate ICB components. A pH-responsive metal-organic framework containing curcumin and the PD-1/PD-L1 inhibitor BMS1166 was developed for osteosarcoma [121]. Curcumin promoted autophagy-associated immunogenic cell death, while BMS1166 blocked checkpoint signaling. The combination increased tumor immunogenicity and improved antitumor T-cell responses [121]. In another design, PD-1-overexpressing engineered nanovesicles were coated onto curcumin-loaded PLGA nanoparticles. The surface PD-1 protein acted as a decoy to interfere with PD-1/PD-L1 interactions, while curcumin induced ICD. The resulting formulation increased mature DCs and CD8^+^ T cells in breast and prostate tumor models [122]. These studies are useful examples of nano-enabled immunotherapy, although their direct metabolic evidence is weaker than that of glucose-, ferroptosis-, or cuproptosis-oriented systems.

Vascular and hypoxic remodeling represents another way to improve ICB responsiveness. Gambogic acid-loaded biomimetic nanoparticles coated with 4T1 tumor-cell membranes were designed to combine photothermal therapy with vascular normalization [123]. Gambogic acid inhibited HIF-1α and VEGF signaling, reduced hypoxic stress, and improved immune-cell infiltration. The nanoplatform induced ICD, activated dendritic cells and cytotoxic T cells, and reduced Tregs. Anti-PD-L1 further amplified the antitumor effect and suppressed lung metastasis. This study is relevant because hypoxia is itself a major metabolic constraint on antitumor immunity, so improving perfusion and oxygen availability can indirectly support checkpoint therapy.

Honokiol-based nanomedicine offers a related example involving the myeloid compartment. Liposomal honokiol inhibited M2 macrophage polarization in non-small cell lung cancer and enhanced the response to PD-1 blockade [124]. The formulation increased the proportion and activity of antitumor immune populations while reducing the suppressive macrophage phenotype. Although direct metabolic measurements in TAMs were not central to this study, it supports the broader idea that natural product nanomedicines can improve ICB by changing the cellular context in which immune checkpoint blockade operates.

Chlorogenic acid has also been tested in combination with PD-1 blockade. Sialic-acid-modified chlorogenic acid liposomes reduced M2-like TAMs and FoxP3^+^ Tregs while increasing M1-like TAMs and CD8^+^ T cells in melanoma. Combination with anti-PD-1 produced stronger tumor inhibition than either treatment alone [125]. Although this study demonstrated changes in immune-cell populations and improved antitumor efficacy, it did not directly establish metabolic remodeling and is therefore better regarded as supportive evidence for natural product nano-immunotherapy rather than a confirmed immunometabolic mechanism.

Taken together, current studies indicate that natural product nanomedicines can enhance ICB through several mechanistically distinct routes. The strongest immunometabolic evidence comes from systems that directly alter PKM2-dependent glycolysis, GSH-dependent redox balance, ferroptosis/cuproptosis, mitochondrial stress, or hypoxia, and then show improved PD-1/PD-L1 therapy. Other nanomedicines primarily induce ICD, regulate checkpoint expression, or remodel TAMs and Tregs without directly measuring metabolic flux. These two levels of evidence should not be conflated. Importantly, greater tumor inhibition with combination therapy does not, by itself, establish ICB sensitization or pharmacological synergy. Stronger evidence requires appropriate resistant or immune-excluded models, demonstration of a defined resistance mechanism being reversed, or formal assessment of treatment interactions. Treatment sequencing and durable immune memory, including rechallenge responses, provide additional evidence of therapeutic benefit where evaluated. Mechanistically, these effects can be further distinguished as direct or indirect immune regulation. Direct regulation involves altering metabolic pathways within immune cells themselves, whereas indirect regulation primarily modifies tumor-cell metabolism, thereby changing nutrient availability, lactate accumulation, or metabolic stress in the tumor microenvironment. Current evidence is more substantial for indirect regulation, while direct immune-cell metabolic reprogramming remains less extensively demonstrated.

**Figure 3:**
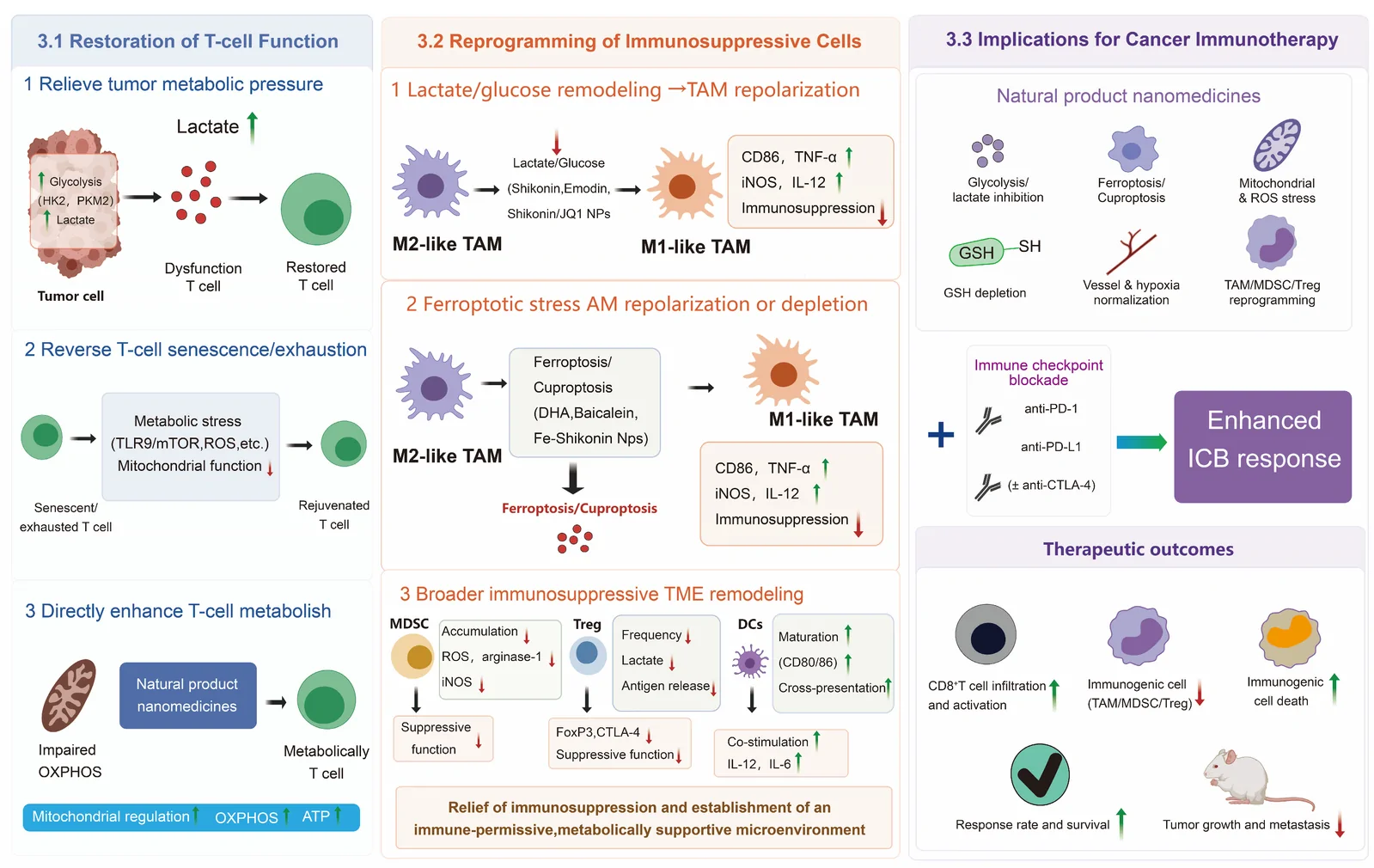
Immunological consequences of metabolic remodeling by natural product nanomedicines. The schematic summarizes potential effects on CD8^+^ T-cell responses, immunosuppressive immune-cell populations, and the efficacy of cancer immunotherapy. Arrows represent proposed relationships between metabolic alterations and immune outcomes, while ↑ and ↓ indicate increases and decreases, respectively. TAMs, tumor-associated macrophages; MDSCs, myeloid-derived suppressor cells; Tregs, regulatory T cells; DCs, dendritic cells; ICB, immune checkpoint blockade.

The available literature also suggests that metabolic remodeling is most useful when it addresses a defined source of checkpoint resistance. Excessive glycolysis can deprive T cells of nutrients, hypoxia limits T-cell infiltration and function, redox buffering can prevent immunogenic metabolic death, and suppressive stromal or myeloid populations can maintain PD-L1 signaling even after checkpoint blockade. Nanomedicine provides a way to localize these interventions and coordinate them temporally with ICB. The next step will be to move beyond simple combination efficacy and identify biomarkers that predict which metabolic vulnerability should be targeted in each tumor type. Such studies would help transform natural product nanomedicines from empirical ICB adjuvants into mechanism-guided immunometabolic therapies. Overall, metabolic remodeling by natural product nanomedicines may restore T-cell function, reprogram immunosuppressive cell populations, and create a tumor immune microenvironment more responsive to cancer immunotherapy (Figure 3). Table 2 summarizes representative natural product nanomedicines combined with cancer immunotherapy and categorizes the available evidence linking metabolic regulation to immune outcomes. Details of the reported mechanistic validation experiments, component-specific controls, and available pharmacokinetic/biodistribution evidence for these studies are provided in Supplementary Table S1.

## 5. Challenges and Perspectives

Despite growing evidence that natural product nanomedicines can alter tumor metabolism and improve antitumor immunity, the field remains at an early mechanistic stage. One of the main limitations is the weak causal linkage between metabolic regulation and immune outcomes. In many studies, changes in lactate, ROS, GSH, lipid peroxidation, or mitochondrial activity are observed alongside increased CD8^+^ T-cell infiltration, TAM repolarization, or improved checkpoint blockade. These parallel changes do not necessarily prove that the immune response is driven by the metabolic intervention itself. Recent work in tumor immunometabolism has emphasized that metabolic pathways act through highly interconnected networks rather than isolated linear axes [5,7]. This complexity becomes even more relevant when several metabolic programs are altered simultaneously by a multifunctional nanoplatform. Rescue experiments using exogenous metabolites, pathway-specific inhibitors, genetic perturbation, or cell-selective metabolic blockade should be incorporated more routinely. Such approaches would help distinguish direct immunometabolic regulation from secondary immune changes caused by tumor regression or cell death. Similarly, increased ROS, GSH depletion, reduced GPX4 expression, or lipid peroxidation alone cannot definitively establish ferroptosis or other regulated cell-death mechanisms. Stronger evidence requires pathway-specific inhibition or rescue experiments. Claims of immunogenic cell death should also be supported by appropriate immunogenicity markers, such as calreticulin exposure, extracellular ATP release, and HMGB1 release, with vaccination or tumor rechallenge experiments providing further functional evidence where available.

This issue also affects the interpretation of metabolic cell death. Ferroptosis and related redox-dependent processes are now widely exploited in cancer nanomedicine, yet their immune consequences depend strongly on the cellular context [23,126]. Lipid peroxidation in malignant cells may promote immunogenic stress and improve treatment responses, while similar oxidative damage in activated lymphocytes can compromise their survival and effector function. The objective should therefore not be maximal metabolic disruption throughout the tumor microenvironment. A more appropriate goal is to generate metabolic asymmetry, in which tumor cells or selected suppressive populations experience severe metabolic stress while cytotoxic and memory T cells retain sufficient metabolic fitness. Cell-selective uptake, stimulus-responsive release, organelle targeting, and controlled exposure will be central to achieving this balance.

Current studies also remain strongly tumor-cell centered. Most natural product nanomedicines are designed to inhibit tumor glycolysis, induce ferroptosis, deplete GSH, or damage mitochondria. Far fewer systems directly restore the metabolic capacity of immune cells. This imbalance is important because effective immunotherapy depends on metabolically competent lymphocytes. Recent studies and reviews have increasingly linked T-cell exhaustion, senescence, and immunotherapy resistance with defects in mitochondrial respiration, nutrient utilization, and metabolic flexibility [127,128]. Directly improving CD8^+^ T-cell oxidative phosphorylation, mitochondrial biogenesis, redox resilience, or amino-acid utilization may eventually be as important as suppressing tumor-cell metabolism. The few examples discussed above that directly target T-cell metabolic fitness suggest that this approach is feasible, but it remains underdeveloped.

Another challenge is the pronounced metabolic heterogeneity within and between tumors. Glucose dependence, lactate production, amino-acid utilization, lipid handling, hypoxia, and mitochondrial activity vary across tumor regions and cell populations. The same metabolic target may therefore have very different biological consequences in different tumors. Recent work has also broadened the concept of tumor metabolism beyond the local TME, showing that systemic host metabolism can influence nutrient availability, immune-cell function, and treatment response [129]. This raises a practical concern for natural product nanomedicines: a formulation optimized for one tumor model may not engage the same metabolic vulnerability in another patient or even in another region of the same tumor. Biomarker-guided patient selection and metabolic phenotyping will likely be required before these strategies can be translated into precision immunotherapy.

Technical advances in spatial and single-cell analysis may help address this problem. Conventional bulk metabolomics provides useful information on global metabolic changes but cannot determine which cell population is responsible for them. In contrast, emerging methods now combine mass spectrometry imaging with high-dimensional immune phenotyping, allowing metabolic states to be mapped to specific cell populations within intact tissues [130]. Cross-modal platforms integrating spatial transcriptomics and spatial metabolomics are also becoming available and can reveal how transcriptional programs, metabolites, and tissue architecture interact within the same tumor [131]. Spatial mapping of tumor immune ecosystems has already demonstrated substantial regional heterogeneity in immune organization and functional states [132]. Applying these approaches to nanomedicine studies would make it possible to ask a much more precise question: which metabolic pathway is changed, in which cell type, and in which region of the tumor after treatment?

The pharmaceutical properties of natural products add another layer of complexity. Nanocarriers can improve solubility, circulation, and tissue exposure, but the pharmacological activity of a natural compound may change after encapsulation, conjugation, or metal coordination. Drug release kinetics, chemical stability, active metabolite formation, and carrier-drug interactions are not always characterized in sufficient depth. A nanoformulation should therefore not be assumed to reproduce the metabolic activity of the free compound. The studies reviewed in Section 3 and Section 4 illustrate this point clearly: only a subset of nanoformulations directly measured the metabolic endpoint attributed to the natural product. Future work should establish an explicit exposure-mechanism relationship by linking intratumoral drug concentration with metabolic target engagement and immune response.

An additional challenge in interpreting the immunometabolic effects of natural products arises from their intrinsic chemical reactivity and potential interference with biochemical assays. Compounds such as shikonin, celastrol, and dihydroartemisinin possess electrophilic and/or redox-active structural features that may contribute to broader cellular effects beyond specific metabolic target regulation. Consequently, changes in GSH levels, ROS production, GPX4 expression, or lipid peroxidation should not, by themselves, be considered definitive evidence of direct target engagement. Moreover, potential interference with commonly used colorimetric and fluorescence-based assays should be considered, particularly when redox-active compounds or metal-containing nanocarriers are involved. Future studies should employ complementary approaches, including cellular thermal shift assays, purified-protein enzymatic assays, genetic perturbation, and orthogonal biochemical measurements, to distinguish target-specific metabolic regulation from nonspecific chemical effects and assay-related artifacts.

Formulation reproducibility is equally important. Complex systems containing multiple lipids, polymers, metal ions, biomimetic membranes, targeting ligands, or natural compounds may perform well at laboratory scale but become difficult to reproduce during scale-up. Critical quality attributes such as particle size distribution, drug loading, release rate, surface chemistry, aggregation, and colloidal stability can alter biodistribution and biological activity. Recent translational frameworks for nanomedicine emphasize that developability, manufacturability, scalability, and regulatory planning should be considered early rather than after efficacy has been demonstrated [37,133]. Quality-by-design approaches may help reduce batch variability by defining critical material attributes and process parameters during formulation development [134].

Biological identity represents another source of variability. Once nanoparticles enter the circulation, adsorbed proteins generate a dynamic protein corona that can change cellular uptake, clearance, organ distribution, and targeting efficiency. These effects vary with particle composition and also with patient-specific factors such as disease state and serum composition [135]. A targeting ligand that performs well in a simplified in vitro system may therefore behave differently in vivo. Recent work further shows that protein corona composition and PEGylation can profoundly alter nanoparticle accumulation and therapeutic activity in cancer models [136]. More realistic evaluation using patient-derived serum, organoid models, and heterogeneous tumor systems may improve prediction of clinical behavior.

Tumor penetration remains a related obstacle. Metabolic targets are often distributed throughout poorly perfused and hypoxic tumor regions, yet many nanoparticles accumulate mainly around tumor vasculature. Improving total tumor accumulation does not necessarily guarantee access to the cells that maintain metabolic resistance. Strategies that address extracellular matrix barriers, vascular abnormalities, particle size, charge, and transcytosis may therefore be necessary for effective immunometabolic modulation [137]. This is particularly important when the intended target is an immune or stromal population rather than the malignant cell itself.

Another important challenge is distinguishing the contribution of natural products from that of other active components within multifunctional nanoplatforms. Metal ions, metal-organic frameworks, photothermal agents, siRNA, and immune checkpoint inhibitors may independently influence redox homeostasis, regulated cell death, or immune signaling. Therefore, the biological effects of a complete formulation should not automatically be attributed to the natural product alone. Where available, comparisons with free drugs, empty carriers, individual active components, and appropriate combination controls are essential for identifying their respective contributions. In the absence of such evidence, the observed effects should be interpreted as those of the combined nanoplatform rather than as direct consequences of natural product-mediated metabolic regulation.

Safety also requires more careful consideration. Metabolic pathways are shared by tumor cells, immune cells, and normal tissues. Glycolysis, mitochondrial respiration, GSH-dependent redox regulation, amino-acid metabolism, and lipid oxidation are fundamental physiological processes. Systemic or prolonged interference may therefore produce effects that are not apparent in short-term tumor models. The risk is amplified when nanocarriers prolong drug exposure or concentrate redox-active compounds within organs of clearance. Repeated-dose toxicity, immune toxicity, mitochondrial injury, hematological effects, and long-term metabolic consequences should be evaluated alongside tumor inhibition. The recent clinical experience with approved natural product-derived nanomedicines confirms that translation is possible, but also highlights the importance of formulation-specific pharmacology and safety assessment [32].

A further limitation is the weak connection between pharmacokinetics and pharmacodynamics in many preclinical studies. Tumor accumulation is often reported as fluorescence intensity, whereas target engagement is assessed separately by Western blotting or flow cytometry. These measurements rarely establish whether drug exposure is sufficient to produce a defined metabolic change in the relevant cell population. For example, a shikonin-loaded nanoplatform (SHK@HA-MPDA) exhibited an IC_50_ of approximately 0.30 μM in CT26 colorectal cancer cells [72]. However, this cellular IC_50_ does not establish the intratumoral concentration of pharmacologically available shikonin or its direct inhibition of PKM2 in vivo. Similar exposure-response uncertainties remain for celastrol-based nanoformulations, despite reported improvements in drug delivery and antitumor activity. Future studies should integrate quantitative plasma and tumor pharmacokinetics with metabolomics, immune profiling, and dose-response analysis, particularly by comparing intratumoral drug exposure with experimentally established effective concentrations. Such a framework would allow investigators to distinguish delivery failure from biological resistance and would provide a more rational basis for selecting doses and combination schedules.

The interpretation of immunological outcomes also depends on the experimental tumor model. Immunocompetent syngeneic models allow evaluation of antitumor immune responses in the presence of an intact murine immune system, whereas immune-deficient xenograft models are less suitable for establishing adaptive immune mechanisms. Orthotopic and metastatic models provide additional information about tumor location and disease progression, while humanized models offer opportunities to investigate human immune components but have their own limitations. Therefore, the model type and immune status should be considered when comparing the immunological evidence across studies.

The next stage of the field should therefore move from descriptive metabolic remodeling toward mechanism-guided, cell-resolved, and pharmacologically defined immunometabolic intervention. Spatial metabolomics and single-cell technologies can identify relevant metabolic vulnerabilities; nanomedicine can provide selective delivery; and pharmacokinetic-pharmacodynamic modeling can determine whether sufficient target engagement has been achieved. These components should be integrated rather than studied separately. Ultimately, the most informative studies will be those that can answer four questions in the same experimental system: what metabolic process changed, in which cell population, at what drug exposure, and whether that change was responsible for the immune and therapeutic response. Meeting these requirements will be essential if natural product nanomedicines are to progress from proof-of-concept metabolic modulators to reproducible and clinically useful partners for cancer immunotherapy.

## 6. Conclusions

Tumor metabolism is closely intertwined with antitumor immunity. Competition for glucose, lactate accumulation, altered lipid and amino acid metabolism, and mitochondrial and redox dysfunction can collectively create an environment in which cytotoxic lymphocytes lose function while suppressive immune populations are maintained. Natural products can act on many of these metabolic processes, but their therapeutic use is often limited by poor solubility, rapid clearance, insufficient tumor exposure, and lack of cellular selectivity. Nanomedicine provides a practical means of overcoming some of these limitations and, more importantly, enables metabolic interventions to be directed toward specific tumor compartments.

Current studies show that natural product nanomedicines can suppress glycolysis and lactate production, disturb lipid and redox homeostasis, trigger ferroptotic or cuproptotic stress, and alter mitochondrial function. These metabolic changes can translate into increased CD8^+^ T-cell activity, TAM repolarization, reduced MDSC or Treg accumulation, enhanced immunogenic cell death, and improved responses to immune checkpoint blockade. The evidence, however, is uneven. Direct metabolic reprogramming of immune cells remains relatively uncommon, and many studies demonstrate metabolic and immune changes in parallel without establishing a causal relationship between them. The distinction between tumor-cell metabolic disruption and genuine immune-cell metabolic restoration therefore needs to remain clear.

Future development should place greater emphasis on cell-specific metabolic targeting, direct measurement of immune-cell metabolism, and pharmacokinetic-pharmacodynamic relationships. Spatial metabolomics and single-cell approaches may help identify which metabolic pathways are altered in individual tumor and immune populations, while better-defined nanoformulations will be needed to translate these findings into reproducible therapies. The field is moving beyond the use of nanocarriers simply to improve natural product delivery. A more compelling direction is to engineer natural product nanomedicines that produce controlled and cell-selective metabolic changes capable of relieving immunosuppression and improving cancer immunotherapy. Establishing the causal links among drug exposure, metabolic target engagement, immune remodeling, and therapeutic response will be critical for this transition.

## Figures and Tables

**Table 1:** Representative Natural Product Nanomedicines for Tumor Immuno-metabolic Modulation.

Natural Product	Nanoplatform	Cancer Model	Target Cell Type	Metabolic Target and Direct Measurement	Immune Outcome	Mechanistic Validation	Ref.
Shikonin	HA-modified mesoporous polydopamine nanoparticles	Colorectal cancer liver metastasis	Tumor cells; dendritic cells; NK cells; CD8^+^ T cells; MDSCs	PKM2-dependent glycolysis inhibition; decreased PKM2 expression; reduced lactate production and ATP generation; reduced intratumoral lactate levels	Reduced MDSC recruitment; increased NK cells and CD8^+^ T cells; increased granzyme B and IFN-γ; enhanced DC activation	Lactate-associated immunosuppression was functionally linked to immune remodeling; lactate reduction contributed to decreased MDSC migration	[72]
Resveratrol	PEG-PLA polymeric nanoparticles	CT26 colorectal cancer	Tumor cells	Glucose metabolism inhibition; reduced glucose utilization and decreased 18F-FDG uptake in vitro and in vivo	Not evaluated	Not performed for immune regulation	[74]
Resveratrol	Dual-responsive polyplex co-delivering PD-L1 siRNA	Breast cancer	Tumor cells; T cells; immunosuppressive immune cells	Reduced glucose consumption and lactate production; suppressed glycolysis; metabolic shift toward OXPHOS; regulated PKM2/AMPK axis	Enhanced antitumor immunity through PD-L1 silencing; improved T-cell-mediated immune response	Combined metabolic regulation with PD-L1 pathway inhibition	[75]
Dihydroartemisinin	ZnP core-shell nanoparticles co-delivering Chol-DHA and Pyro-Fe	Colorectal cancer	Tumor cells; immune cells	Iron-dependent ferroptosis; increased ROS production; enhanced lipid peroxidation; ferroptotic cell death	Increased tumor immunogenicity; enhanced CRT exposure and HMGB1 release; increased intratumoral CD8^+^ T-cell infiltration; enhanced anti-PD-L1 therapy	Co-delivery of DHA and Pyro-Fe increased ferroptotic immunogenicity and sensitized tumors to checkpoint blockade	[78]
Dihydroartemisinin	Fe-MnO_2_ nanosheets	Hepatocellular carcinoma	Tumor cells; immune cells (T cells; macrophages)	GSH/GPX4 axis regulation; decreased GPX4 activity; increased lipid peroxidation and ferroptosis-related oxidative stress	Induced immunogenic cell death; increased CD4^+^ and CD8^+^ T-cell infiltration; promoted macrophage polarization; enhanced antitumor immune response	Ferroptosis inhibition experiments and evaluation of GPX4/GSH/lipid peroxide changes validated the ferroptotic mechanism	[80]
Dihydroartemisinin	DHA-loaded MIL-101 nanoreactor	Tumor model/TAMs	Tumor-associated macrophages	Iron-dependent ferroptosis; ferroptotic stress induction	Activation of ferroptosis-associated cGAS-STING signaling; macrophage immune modulation	Ferroptosis-associated immune signaling validation	[82]
Dihydroartemisinin	Biomimetic ferroptosis nanoinducer	Tumor model	Tumor cells; M2-type TAMs	GSH depletion; Fe^3+^/Fe^2+^ conversion; increased Fe^2+^-dependent oxidative stress; ferroptosis induction	Reprogramming of immunosuppressive M2-TAM phenotype; remodeling of tumor immune microenvironment	Ferroptosis inhibition reduced therapeutic effects; TAM-associated ferroptotic regulation was validated	[83]
Shikonin	Shikonin-Fe(III) nanocomplex incorporated in tragacanth gum	Bladder cancer	Tumor cells; dendritic cells; T cells	Iron metabolism and lipid peroxidation; Fe^2+^-dependent oxidative stress; ferroptosis induction	Induction of ICD; enhanced antigen presentation and antitumor immune activation	Ferroptosis inhibition reduced ICD-related effects	[85]
Shikonin	GSH-responsive Fe-shikonin nanomedicine	Head and neck squamous cell carcinoma	Tumor cells; DCs; macrophages; cytotoxic T lymphocytes	GSH depletion; Fe^2+^ generation; lipid peroxidation; ferroptosis induction	Enhanced ICD; promoted DC maturation and antigen cross-presentation; increased CTL infiltration; immune memory induction	GSH-responsive Fe^2+^ release and ferroptosis/ICD pathway validation	[86]
Celastrol	ATP-responsive mitochondria-targeted metal-polyphenol network	Postoperative tumor model	Tumor cells; innate immune cells	Mitochondrial membrane disruption; mtDNA leakage; mitochondrial stress	Activation of innate immune sensing pathways	Mitochondrial injury and mtDNA-mediated immune activation validation	[95]

**Table 2:** Representative Natural Product Nanomedicines Combined with Cancer Immunotherapy.

Natural Product Nanoplatform	Tumor Model	Immunotherapy Strategy	Metabolic Target and Mechanistic Evidence	Immune-Related Outcomes	Evidence Category	Ref.
Emodin-loaded biomimetic iron-based nanoparticles	4T1 breast cancer (subcutaneous, BALB/c, immunocompetent)	Metabolic immunotherapy	Reduced lactate production and increased tumor iron accumulation; lactate-associated TAM repolarization was observed	Increased M1-like TAM markers (CD16/32) and pro-inflammatory cytokine production; reduced immunosuppressive features	II	[107]
Mannosylated lactoferrin nanoparticles co-delivering shikonin and JQ1	CT26 colorectal cancer (subcutaneous, immunocompetent)	JQ1-mediated PD-L1 suppression	Shikonin-associated glycolytic suppression and reduced glucose metabolism; tumor-cell death and immunogenic-cell-death-related effects were reported	Increased DC maturation and CD8^+^ T-cell infiltration; reduced Tregs; altered TAM polarization	II	[108]
Dihydroartemisinin-containing composite nanohydrogel	Orthotopic 4T1 breast cancer (BALB/c, immunocompetent)	Anti-PD-1 blockade	Copper chelator interventions using TTM and D-penicillamine partially rescued treatment-induced cell death; copper-related protein changes and DLAT oligomerization supported cuproptosis	Increased DAMP-associated signals, intratumoral CD8^+^ T cells and antitumor immune responses	III	[116]
Glutathione-scavenging celastrol-Cu nanoparticles	4T1 breast cancer (subcutaneous, BALB/c, immunocompetent)	Immune checkpoint blockade	GSH depletion and copper accumulation support the proposed cuproptosis mechanism; ICD-related immune activation was reported	Enhanced antitumor immune responses and control of metastatic tumors	III	[117]
Curcumin-loaded copper-based nanocomposites	AKR esophageal carcinoma (subcutaneous, male C57 mice, immunocompetent)	Photothermal therapy plus anti-PD-1 blockade	Curcumin-associated EGFR-STAT3 inhibition and PD-L1 downregulation; ROS generation, mitochondrial dysfunction and DAMP release were reported following combined treatment	Increased cytotoxic T-cell infiltration and DC maturation; reduced Tregs	III	[120]

Footnotes: Evidence category I indicates direct metabolic regulation of immune cells; category II indicates tumor-cell metabolic alterations associated with indirect immune benefits; category III indicates metabolic stress or regulated cell death accompanied by immune activation; and category IV indicates immunomodulatory effects without direct evidence of metabolic involvement.

## Data Availability

No new data were created or analyzed in this study. Data sharing is not applicable to this article.

## Supplementary Materials

The following supporting information can be downloaded at: https://www.mdpi.com/article/10.3390/molecules31213965/s1, Table S1: Mechanistic Validation and Supporting Evidence.

## Abbreviations

The following abbreviations are used in this manuscript:

ARG1	Arginase 1
DCs	Dendritic cells
DHA	Dihydroartemisinin
GSH	Glutathione
GPX4	Glutathione peroxidase 4
ICB	Immune checkpoint blockade
ICD	Immunogenic cell death
IDO1	Indoleamine 2,3-dioxygenase 1
LDHA	Lactate dehydrogenase A
MDSCs	Myeloid-derived suppressor cells
MOF	Metal-organic framework
OXPHOS	Oxidative phosphorylation
PKM2	Pyruvate kinase M2
ROS	Reactive oxygen species
TAMs	Tumor-associated macrophages
TDO	Tryptophan 2,3-dioxygenase
TME	Tumor microenvironment
Tregs	Regulatory T cells

## References

[1] Oliveira G., Wu C.J. (2023). Dynamics and specificities of T cells in cancer immunotherapy. Nat. Rev. Cancer.

[2] Schenkel J.M., Pauken K.E. (2023). Localization, tissue biology and T cell state—Implications for cancer immunotherapy. Nat. Rev. Immunol..

[3] Roerden M., Spranger S. (2025). Cancer immune evasion, immunoediting and intratumour heterogeneity. Nat. Rev. Immunol..

[4] Grippin A.J., Lee D., Parkes E.E., Jiang W., Kim B.Y.S. (2025). Nanotechnology for immuno-oncology. Nat. Cancer.

[5] De Martino M., Rathmell J.C., Galluzzi L., Vanpouille-Box C. (2024). Cancer cell metabolism and antitumour immunity. Nat. Rev. Immunol..

[6] Dang Q., Li B., Jin B., Ye Z., Lou X., Wang T., Wang Y., Pan X., Hu Q., Li Z. (2024). Cancer immunometabolism: Advent, challenges, and perspective. Mol. Cancer.

[7] Chapman N.M., Chi H. (2024). Metabolic rewiring and communication in cancer immunity. Cell Chem. Biol..

[8] Liu Y., Zhao Y., Song H., Li Y., Liu Z., Ye Z., Zhao J., Wu Y., Tang J., Yao M. (2024). Metabolic reprogramming in tumor immune microenvironment: Impact on immune cell function and therapeutic implications. Cancer Lett..

[9] Demicco M., Liu X.-Z., Leithner K., Fendt S.-M. (2024). Metabolic heterogeneity in cancer. Nat. Metab..

[10] Trefny M.P., Kroemer G., Zitvogel L., Kobold S. (2025). Metabolites as agents and targets for cancer immunotherapy. Nat. Rev. Drug Discov..

[11] Zhang S., Zhang X., Yang H., Liang T., Bai X. (2024). Hurdle or thruster: Glucose metabolism of T cells in anti-tumour immunity. Biochim. Biophys. Acta (BBA) Rev. Cancer.

[12] Liang X.-H., Chen X.-Y., Yan Y., Cheng A.-Y., Lin J.-Y., Jiang Y.-X., Chen H.-Z., Jin J.-M., Luan X. (2024). Targeting metabolism to enhance immunotherapy within tumor microenvironment. Acta Pharmacol. Sin..

[13] Su R., Shao Y., Huang M., Liu D., Yu H., Qiu Y. (2024). Immunometabolism in cancer: Basic mechanisms and new targeting strategy. Cell Death Discov..

[14] Dong C., Zhao Y., Han Y., Li M., Wang G. (2025). Targeting glutamine metabolism crosstalk with tumor immune response. Biochim. Biophys. Acta (BBA) Rev. Cancer.

[15] Chen J., Cui L., Lu S., Xu S. (2024). Amino acid metabolism in tumor biology and therapy. Cell Death Dis..

[16] Grobben Y. (2024). Targeting amino acid-metabolizing enzymes for cancer immunotherapy. Front. Immunol..

[17] Wu Y., Pu X., Wang X., Xu M. (2024). Reprogramming of lipid metabolism in the tumor microenvironment: A strategy for tumor immunotherapy. Lipids Health Dis..

[18] Taranto D., Kloosterman D.J., Akkari L. (2024). Macrophages and T cells in metabolic disorder-associated cancers. Nat. Rev. Cancer.

[19] Wang L., Zhang J., Zhang W., Zheng M., Guo H., Pan X., Li W., Yang B., Ding L. (2024). The inhibitory effect of adenosine on tumor adaptive immunity and intervention strategies. Acta Pharm. Sin. B.

[20] Yang H., Zhang Z., Zhao K., Zhang Y., Yin X., Zhu G., Wang Z., Yan X., Li X., He T. (2024). Targeting the adenosine signaling pathway in macrophages for cancer immunotherapy. Hum. Immunol..

[21] Ma S., Ming Y., Wu J., Cui G. (2024). Cellular metabolism regulates the differentiation and function of T-cell subsets. Cell. Mol. Immunol..

[22] Feng B., Li R., Li W., Tang L. (2024). Metabolic immunoengineering approaches to enhance CD8+ T cell-based cancer immunotherapy. Cell Syst..

[23] Nakamura T., Conrad M. (2024). Exploiting ferroptosis vulnerabilities in cancer. Nat. Cell Biol..

[24] Zhai X., Lin Y., Zhu L., Wang Y., Zhang J., Liu J., Li L., Lu X. (2024). Ferroptosis in cancer immunity and immunotherapy: Multifaceted interplay and clinical implications. Cytokine Growth Factor. Rev..

[25] Nguyen L.N.M., Ngo W., Lin Z.P., Sindhwani S., MacMillan P., Mladjenovic S.M., Chan W.C.W. (2024). The mechanisms of nanoparticle delivery to solid tumours. Nat. Rev. Bioeng..

[26] Wang J., Yang Y., Shao F., Meng Y., Guo D., He J., Lu Z. (2024). Acetate reprogrammes tumour metabolism and promotes PD-L1 expression and immune evasion by upregulating c-Myc. Nat. Metab..

[27] Zhou Q., Meng Y., Li D., Yao L., Le J., Liu Y., Sun Y., Zeng F., Chen X., Deng G. (2024). Ferroptosis in cancer: From molecular mechanisms to therapeutic strategies. Signal Transduct. Target. Ther..

[28] Fakhri S., Moradi S.Z., Moradi S.Y., Piri S., Shiri Varnamkhasti B., Piri S., Khirehgesh M.R., Bishayee A., Casarcia N., Bishayee A. (2024). Phytochemicals regulate cancer metabolism through modulation of the AMPK/PGC-1α signaling pathway. BMC Cancer.

[29] Mahato R., Behera D.k., Patra B., Das S., Lakra K., Pradhan S.N., Abbas S.J., Ali S.I. (2024). Plant-based natural products in cancer therapeutics. J. Drug Target..

[30] Biswal S., Sahoo S.K., Biswal B.K. (2025). Shikonin a potent phytotherapeutic: A comprehensive review on metabolic reprogramming to overcome drug resistance in cancer. Mol. Biol. Rep..

[31] Andreani T., Cheng R., Elbadri K., Ferro C., Menezes T., Dos Santos M.R., Pereira C.M., Santos H.A. (2024). Natural compounds-based nanomedicines for cancer treatment: Future directions and challenges. Drug Deliv. Transl. Res..

[32] Xiaotong L.I., Yaoyao L.A.I., Guanghan W.A.N., Jiahui Z.O.U., Wei H.E., Pei Y. (2024). Approved natural products-derived nanomedicines for disease treatment. Chin. J. Nat. Med..

[33] Hadjidemetriou M., Mahmoudi M., Kostarelos K. (2024). In vivo biomolecule corona and the transformation of a foe into an ally for nanomedicine. Nat. Rev. Mater..

[34] Liu J., Bai Y., Li Y., Li X., Luo K. (2024). Reprogramming the immunosuppressive tumor microenvironment through nanomedicine: An immunometabolism perspective. EBioMedicine.

[35] Zeng Y., Xu G., Kong X., Ye G., Guo J., Lu C., Nezamzadeh-Ejhieh A., Khan M.S., Liu J., Peng Y. (2022). Recent advances of the core-shell MOFs in tumour therapy. Int. J. Pharm..

[36] Linderman S.W., Deridder L., Sanjurjo L., Foote M.B., Alonso M.J., Kirtane A.R., Langer R., Traverso G. (2025). Enhancing immunotherapy with tumour-responsive nanomaterials. Nat. Rev. Clin. Oncol..

[37] Joyce P., Allen C.J., Alonso M.J., Ashford M., Bradbury M.S., Germain M., Kavallaris M., Langer R., Lammers T., Peracchia M.T. (2024). A translational framework to DELIVER nanomedicines to the clinic. Nat. Nanotechnol..

[38] Xu N., Zhu Y., Han Y., Liu Q., Tong L., Li Y., Chen Z., Shao S., He W., Li M. (2025). Targeting MondoA-TXNIP restores antitumour immunity in lactic-acid-induced immunosuppressive microenvironment. Nat. Metab..

[39] Niveau C., Cettour-Cave M., Mouret S., Sosa Cuevas E., Pezet M., Roubinet B., Gil H., De Fraipont F., Landemarre L., Charles J. (2025). MCT1 lactate transporter blockade re-invigorates anti-tumor immunity through metabolic rewiring of dendritic cells in melanoma. Nat. Commun..

[40] Chang C.-H., Qiu J., O’Sullivan D., Buck M.D., Noguchi T., Curtis J.D., Chen Q., Gindin M., Gubin M.M., Van Der Windt G.J.W. (2015). Metabolic competition in the tumor microenvironment is a driver of cancer progression. Cell.

[41] Ho P.-C., Bihuniak J.D., Macintyre A.N., Staron M., Liu X., Amezquita R., Tsui Y.-C., Cui G., Micevic G., Perales J.C. (2015). Phosphoenolpyruvate is a metabolic checkpoint of anti-tumor T cell responses. Cell.

[42] Brand A., Singer K., Koehl G.E., Kolitzus M., Schoenhammer G., Thiel A., Matos C., Bruss C., Klobuch S., Peter K. (2016). LDHA-associated lactic acid production blunts tumor immunosurveillance by T and NK cells. Cell Metab..

[43] Colegio O.R., Chu N.-Q., Szabo A.L., Chu T., Rhebergen A.M., Jairam V., Cyrus N., Brokowski C.E., Eisenbarth S.C., Phillips G.M. (2014). Functional polarization of tumour-associated macrophages by tumour-derived lactic acid. Nature.

[44] Chen P., Zuo H., Xiong H., Kolar M.J., Chu Q., Saghatelian A., Siegwart D.J., Wan Y. (2017). Gpr132 sensing of lactate mediates tumor-macrophage interplay to promote breast cancer metastasis. Proc. Natl. Acad. Sci. USA.

[45] Angelin A., Gil-de-Gómez L., Dahiya S., Jiao J., Guo L., Levine M.H., Wang Z., Quinn W.J., Kopinski P.K., Wang L. (2017). Foxp3 reprograms T cell metabolism to function in low-glucose, high-lactate environments. Cell Metab..

[46] Watson M.J., Vignali P.D.A., Mullett S.J., Overacre-Delgoffe A.E., Peralta R.M., Grebinoski S., Menk A.V., Rittenhouse N.L., DePeaux K., Whetstone R.D. (2021). Metabolic support of tumour-infiltrating regulatory T cells by lactic acid. Nature.

[47] Kumagai S., Koyama S., Itahashi K., Tanegashima T., Lin Y.-t., Togashi Y., Kamada T., Irie T., Okumura G., Kono H. (2022). Lactic acid promotes PD-1 expression in regulatory T cells in highly glycolytic tumor microenvironments. Cancer Cell.

[48] Viel S., Vivier E., Walzer T., Marçais A. (2025). Targeting metabolic dysfunction of CD8 T cells and natural killer cells in cancer. Nat. Rev. Drug Discov..

[49] Sung Y., Kim D.K., Kim J.S., Kim S.-J., Kim J.H., Han J.M. (2026). Metabolic networks in the tumor microenvironment: Roles of amino acid and lipid metabolism pathways in cancer progression and therapy. Exp. Mol. Med..

[50] Ma X., Bi E., Lu Y., Su P., Huang C., Liu L., Wang Q., Yang M., Kalady M.F., Qian J. (2019). Cholesterol induces CD8^+^ T cell exhaustion in the tumor microenvironment. Cell Metab..

[51] Tang Y., Chen Z., Zuo Q., Kang Y. (2024). Regulation of CD8^+^ T cells by lipid metabolism in cancer progression. Cell. Mol. Immunol..

[52] Veglia F., Tyurin V.A., Blasi M., De Leo A., Kossenkov A.V., Donthireddy L., To T.K.J., Schug Z., Basu S., Wang F. (2019). Fatty acid transport protein 2 reprograms neutrophils in cancer. Nature.

[53] Ma X., Xiao L., Liu L., Ye L., Su P., Bi E., Wang Q., Yang M., Qian J., Yi Q. (2021). CD36-mediated ferroptosis dampens intratumoral CD8^+^ T cell effector function and impairs their antitumor ability. Cell Metab..

[54] Lupica-Tondo G.L., Arner E.N., Mogilenko D.A., Voss K. (2024). Immunometabolism of ferroptosis in the tumor microenvironment. Front. Oncol..

[55] Rodriguez P.C., Quiceno D.G., Ochoa A.C. (2007). L-arginine availability regulates T-lymphocyte cell-cycle progression. Blood.

[56] Munn D.H., Mellor A.L. (2016). IDO in the tumor microenvironment: Inflammation, counter-regulation, and tolerance. Trends Immunol..

[57] Wetzel T.J., Erfan S.C., Figueroa L.D., Wheeler L.M., Ananieva E.A. (2023). Crosstalk between arginine, glutamine, and the branched chain amino acid metabolism in the tumor microenvironment. Front. Oncol..

[58] Leone R.D., Zhao L., Englert J.M., Sun I.-M., Oh M.-H., Sun I.-H., Arwood M.L., Bettencourt I.A., Patel C.H., Wen J. (2019). Glutamine blockade induces divergent metabolic programs to overcome tumor immune evasion. Science.

[59] Pretto S., Yu Q., Bourdely P., Trusso Cafarello S., Van Acker H.H., Verelst J., Richiardone E., Vanheer L., Roshanzadeh A., Schneppenheim F. (2025). A functional single-cell metabolic survey identifies Elovl1 as a target to enhance CD8^+^ T cell fitness in solid tumours. Nat. Metab..

[60] Steinert E.M., Furtado Bruza B., Danchine V.D., Grant R.A., Vasan K., Kharel A., Zhang Y., Cui W., Szibor M., Weinberg S.E. (2025). Mitochondrial respiration is necessary for CD8+ T cell proliferation and cell fate. Nat. Immunol..

[61] Scharping N.E., Menk A.V., Moreci R.S., Whetstone R.D., Dadey R.E., Watkins S.C., Ferris R.L., Delgoffe G.M. (2016). The tumor microenvironment represses T cell mitochondrial biogenesis to drive intratumoral T cell metabolic insufficiency and dysfunction. Immunity.

[62] Bengsch B., Johnson A.L., Kurachi M., Odorizzi P.M., Pauken K.E., Attanasio J., Stelekati E., McLane L.M., Paley M.A., Delgoffe G.M. (2016). Bioenergetic insufficiencies due to metabolic alterations regulated by the inhibitory receptor PD-1 are an early driver of CD8+ T cell exhaustion. Immunity.

[63] Buck M.D., O’Sullivan D., Geltink R.I.K., Curtis J.D., Chang C.-H., Sanin D.E., Qiu J., Kretz O., Braas D., van der Windt G.J.W. (2016). Mitochondrial dynamics controls T cell fate through metabolic programming. Cell.

[64] Simula L., Fumagalli M., Vimeux L., Rajnpreht I., Icard P., Birsen G., An D., Pendino F., Rouault A., Bercovici N. (2024). Mitochondrial metabolism sustains CD8+ T cell migration for an efficient infiltration into solid tumors. Nat. Commun..

[65] Ikeda H., Kawase K., Nishi T., Watanabe T., Takenaga K., Inozume T., Ishino T., Aki S., Lin J., Kawashima S. (2025). Immune evasion through mitochondrial transfer in the tumour microenvironment. Nature.

[66] Baldwin J.G., Heuser-Loy C., Saha T., Schelker R.C., Slavkovic-Lukic D., Strieder N., Hernandez-Lopez I., Rana N., Barden M., Mastrogiovanni F. (2024). Intercellular nanotube-mediated mitochondrial transfer enhances T cell metabolic fitness and antitumor efficacy. Cell.

[67] Scharping N.E., Rivadeneira D.B., Menk A.V., Vignali P.D.A., Ford B.R., Rittenhouse N.L., Peralta R., Wang Y., Wang Y., DePeaux K. (2021). Mitochondrial stress induced by continuous stimulation under hypoxia rapidly drives T cell exhaustion. Nat. Immunol..

[68] Jackson C.M., Pant A., Dinalankara W., Choi J., Jain A., Nitta R., Yazigi E., Saleh L., Zhao L., Nirschl T.R. (2024). The cytokine Meteorin-like inhibits anti-tumor CD8+ T cell responses by disrupting mitochondrial function. Immunity.

[69] Van der Windt G.J.W., Everts B., Chang C.-H., Curtis J.D., Freitas T.C., Amiel E., Pearce E.J., Pearce E.L. (2012). Mitochondrial respiratory capacity is a critical regulator of CD8+ T cell memory development. Immunity.

[70] Kawalekar O.U., O’Connor R.S., Fraietta J.A., Guo L., McGettigan S.E., Posey A.D., Patel P.R., Guedan S., Scholler J., Keith B. (2016). Distinct signaling of coreceptors regulates specific metabolism pathways and impacts memory development in CAR T cells. Immunity.

[71] Peralta R.M., Xie B., Lontos K., Nieves-Rosado H., Spahr K., Joshi S., Ford B.R., Quann K., Frisch A.T., Dean V. (2024). Dysfunction of exhausted T cells is enforced by MCT11-mediated lactate metabolism. Nat. Immunol..

[72] Long L., Xiong W., Lin F., Hou J., Chen G., Peng T., He Y., Wang R., Xu Q., Huang Y. (2023). Regulating lactate-related immunometabolism and EMT reversal for colorectal cancer liver metastases using shikonin targeted delivery. J. Exp. Clin. Cancer Res..

[73] Chen L., Zhao D., Ren X., Ren J., Meng X., Fu C., Li X. (2023). Shikonin-loaded hollow Fe-MOF nanoparticles for enhanced microwave thermal therapy. ACS Biomater. Sci. Eng..

[74] Jung K.-H., Lee J.H., Park J.W., Quach C.H.T., Moon S.-H., Cho Y.S., Lee K.-H. (2015). Resveratrol-loaded polymeric nanoparticles suppress glucose metabolism and tumor growth in vitro and in vivo. Int. J. Pharm..

[75] Jia L., Gao Y., Zhou T., Zhao X.-L., Hu H.-Y., Chen D.-W., Qiao M.-X. (2021). Enhanced response to PD-L1 silencing by modulation of TME via balancing glucose metabolism and robust co-delivery of siRNA/Resveratrol with dual-responsive polyplexes. Biomaterials.

[76] Wahida A., Conrad M. (2025). Decoding ferroptosis for cancer therapy. Nat. Rev. Cancer.

[77] Chen R., Wen L., Guo F., He J., Wong K.H., Chen M. (2025). Glutathione-scavenging natural-derived ferroptotic nano-amplifiers strengthen tumor therapy through aggravating iron overload and lipid peroxidation. J. Control. Release.

[78] Han W., Duan X., Ni K., Li Y., Chan C., Lin W. (2022). Co-delivery of dihydroartemisinin and pyropheophorbide-iron elicits ferroptosis to potentiate cancer immunotherapy. Biomaterials.

[79] Deng Y., Huang S., Jiang G., Zhou L., Nezamzadeh-Ejhieh A., Liu J., Zhou Z. (2024). Current status and prospects of MOFs loaded with H2O2-related substances for ferroptosis therapy. RSC Med. Chem..

[80] Huang D., Xu D., Chen W., Wu R., Wen Y., Liu A., Lin L., Lin X., Wang X. (2023). Fe-MnO2 nanosheets loading dihydroartemisinin for ferroptosis and immunotherapy. Biomed. Pharmacother..

[81] Yang H., Liao D., Cai Z., Zhang Y., Nezamzadeh-Ejhieh A., Zheng M., Liu J., Bai Z., Song H. (2023). Current status of Fe-based MOFs in biomedical applications. RSC Med. Chem..

[82] Li L.G., Yang X.X., Xu H.Z., Yu T.T., Li Q.R., Hu J., Peng X.C., Han N., Xu X., Chen N.N. (2023). A dihydroartemisinin-loaded nanoreactor motivates anti-cancer immunotherapy by synergy-induced ferroptosis to activate Cgas/STING for reprogramming of macrophage. Adv. Healthc. Mater..

[83] Chen M., Shen Y., Pu Y., Zhou B., Bing J., Ge M., Zhu Y., Gao S., Wu W., Zhou M. (2023). Biomimetic inducer enabled dual ferroptosis of tumor and M2-type macrophages for enhanced tumor immunotherapy. Biomaterials.

[84] Chen W., Liu M., Yang H., Nezamzadeh-Ejhieh A., Lu C., Pan Y., Liu J., Bai Z. (2023). Recent advances of Fe (III)/Fe (II)-MPNs in biomedical applications. Pharmaceutics.

[85] Zhao K., Hu J., Zhang H., Chen T., Yang S., Wang H., Wu F., Hong H., Zhang G., Zhang C. (2025). A Shikonin-Iron-Tragacanth gum nanocomplex for synergistic treatment of bladder cancer. Chem. Eng. J..

[86] Gao S., Wu Y., Yu Z., Lv M., Liu L., Liu Y., Chen B., Yin W. (2026). Enhancing antitumor immunotherapy in head and neck cancer via ferroptosis-immune cascade activation by Fe-Shikonin nanomedicine. Colloids Surf. B Biointerfaces.

[87] Cao Q., Wu X., Tan J., Xu H., Zhang Y., Hu Y., Chen Y., Zhang S., Tang T. (2025). Targeting GPX4-dependent ferroptosis via a dihydroartemisinin-conjugated cross-linked lipoic acid nanodrug for endometrial carcinoma therapy. Bioconjugate Chem..

[88] Chen J., Liu Y., You Y., Zhou R., Li Y., Zhou X., Chen X., Yang Y., Chen J., Kwan H.Y. (2024). Biotin-decorated celastrol-loaded ZIF-8 nanoparticles induce ferroptosis for colorectal cancer therapy. Mater. Des..

[89] Du H., Xu T., Yu S., Wu S., Zhang J. (2025). Mitochondrial metabolism and cancer therapeutic innovation. Signal Transduct. Target. Ther..

[90] Chen Z., Chen L., Lyu T.D., Weng S., Xie Y., Jin Y., Wu O., Jones M., Kwan K., Makvnadi P. (2024). Targeted mitochondrial nanomaterials in biomedicine: Advances in therapeutic strategies and imaging modalities. Acta Biomater..

[91] Geng Y., Xiang J., Shao S., Tang J., Shen Y. (2022). Mitochondria-targeted polymer-celastrol conjugate with enhanced anticancer efficacy. J. Control. Release.

[92] Qin Y., Wang Z., Wang X., Zhang T., Hu Y., Wang D., Sun H., Zhang L., Zhu Y. (2023). Therapeutic effect of multifunctional celastrol nanoparticles with mitochondrial alkaline drug release in breast cancer. Mater. Today Adv..

[93] Zheng P., Ding B., Jiang Z., Xu W., Li G., Ding J., Chen X. (2021). Ultrasound-augmented mitochondrial calcium ion overload by calcium nanomodulator to induce immunogenic cell death. Nano Lett..

[94] Zhou L., Du Y., Shang Y., Xiang D., Xia X. (2024). A novel triptolide nano-liposome with mitochondrial targeting for treatment of hepatocellular carcinoma. Int. J. Nanomed..

[95] Zhang Z., Tong J., Cao X., Xu W., Qi Z., Kuang X., Huang Y., He Y., Zeng C., Hu M. (2026). ATP-responsive metal-polyphenol networks deliver celastrol to cancer cell mitochondria to activate mtDNA-cGAS-STING axis and boost tumor immunotherapy. Chem. Eng. J..

[96] Ye Y., Zhang X., Tong Q., Xie Q., Gong X., Du S., Gao P., Li Q., Piao J., Xiong Y. (2026). Targeting mitochondrial-ER homeostasis via autophagy inhibition with celastrol-based nanotherapy for triple-negative breast cancer. J. Nanobiotechnology.

[97] Haku Y., Kitaoka K., Ichimaru K., Hirano T., Wang J., Sonomura K., Maruo A., Hirose S., Wang Y., Ito K. (2026). Active aldehydes accelerate CD8+ T cell exhaustion by metabolic alteration in the tumor microenvironment. Nat. Immunol..

[98] Chang H.-C., Tsai C.-Y., Hsu C.-L., Tai T.-S., Cheng M.-L., Chuang Y.-M., Tang H.-Y., Lin K.-J., Chen J.-J., Chang S.-H. (2025). Asparagine deprivation enhances T cell antitumour response in patients via ROS-mediated metabolic and signal adaptations. Nat. Metab..

[99] Wang S., Wang X., Zheng X., Jiang H., Liu L., Ma N., Dong X. (2025). NGR-modified curcumin nanovesicles reverse immunotherapy resistance in triple-negative breast cancer via TLR9 and mTOR pathway modulation. Cell Biol. Toxicol..

[100] Jiang Z., Li Y. (2026). CD93-targeted resveratrol-loaded PLGA nanoparticles remodel CD8^+^ T cell metabolism through AIF-mediated oxidative phosphorylation to overcome lung cancer immunotherapy resistance. J. Nanobiotechnology.

[101] Yang S., Wang J., Tu K., Huang X., Lv L., Peng M., Xu Q., Liu H., Sun Q., Cai L. (2026). AML-Targeted Metal-Polyphenol Nanoplatform Induces Ferroptosis-ICD Cascade for Antitumor Immunity Boosting. Adv. Sci..

[102] Zhou Y., Wang Q., Zhou Z., Peng X., Qu B., Zhang R. (2025). Self-assembled copper chlorogenic acid nanoparticles: Inducing pyroptosis and cuproptosis to activate antitumor immunity. J. Control. Release.

[103] Ruan H., Chen Y., Qian J., Ma X., Xu Y., Deng Y., Pan Y., Kushwaha A., Kumar A., Zheng B. (2026). Advances in metal-organic framework-based drug delivery system in cuproptosis-mediated cancer therapy. Eur. J. Pharm. Biopharm..

[104] Deng W., Wu Y., Yin Y., Wang J. (2026). Honokiol-loaded nanomicelles reprogram senescence and immune evasion in hepatocellular carcinoma via SIRT3-mediated mitochondrial stabilization. Bioeng. Transl. Med..

[105] Cheng X., Dong J., Jain P., Qin S., Miao Y., Liu K., Theja M.L.V., Butch C.J., Wang Y., Lane L.A. (2026). Ultrasound Activated Hybrid-Biomimetic Nanocarriers That Combine Tumor-Confined CRISPR/Cas9 Metabolic Reprogramming and Cuproptosis With Anticancer Macrophage Polarization. Small.

[106] Tang C., He C., Wang D., Guo J., Yin X., Ye H., Wu L., Zhang Y., Zeng S., Zeng X. (2025). Co-delivery of sorafenib and an FSP1 inhibitor triggers dual ferroptosis in tumor cells and immunosuppressive macrophages for enhanced immunotherapy in mouse models of hepatocellular carcinoma. Nat. Commun..

[107] Zhang W., Li L., Wu Y., Li C., Xu Z.a., Zhang N., Wang X., Zhao Y., Zu T., He Q. (2024). Biomimetic iron-based nanoparticles remodel immunosuppressive tumor microenvironment for metabolic immunotherapy. Int. J. Nanomed..

[108] Wang H., Tang Y., Fang Y., Zhang M., Wang H., He Z., Wang B., Xu Q., Huang Y. (2019). Reprogramming tumor immune microenvironment (TIME) and metabolism via biomimetic targeting codelivery of shikonin/JQ1. Nano Lett..

[109] Xiang J., Suo M., Lan J., Ning S., Liu H., Hong W., Liang J., Zheng J., Zhu D., Mo J. (2025). Novel carrier-free nanomedicine for regulating macrophage phenotype to amplify anti-tumor photoimmunotherapy. Small.

[110] Feng W., Shi W., Cui Y., Xu J., Liu S., Gao H., Zhu S., Liu Y., Zhang H. (2023). Fe (III)-Shikonin supramolecular nanomedicines as immunogenic cell death stimulants and multifunctional immunoadjuvants for tumor vaccination. Theranostics.

[111] Wu L., Pi W., Huang X., Yang L., Zhang X., Lu J., Yao S., Lin X., Tan X., Wang Z. (2025). Orchestrated metal-coordinated carrier-free celastrol hydrogel intensifies T cell activation and regulates response to immune checkpoint blockade for synergistic chemo-immunotherapy. Biomaterials.

[112] Dai Y., Guo Z., Leng D., Jiao G., Chen K., Fu M., Liu Y., Shen Q., Wang Q., Zhu L. (2024). Metal-Coordinated NIR-II Nanoadjuvants with Nanobody Conjugation for Potentiating Immunotherapy by Tumor Metabolism Reprogramming. Adv. Sci..

[113] Tan Y., Xu Q., Chen Y., Liu Y., Liu C., Cui X., Zhao Y. (2025). Ultrasound-activated miR-195-5p/shikonin nanobubbles remodel immunosuppressive microenvironment via immunogenic cell death to potentiate PD-1/PD-L1 blockade in hepatocellular carcinoma. Mater. Today Bio.

[114] Wang D., Nie T., Fang Y., Zhang L., Yu C., Yang M., Du R., Liu J., Zhang L., Feng L. (2025). Tailored Liposomal Nanomedicine Suppresses Incomplete Radiofrequency Ablation-Induced Tumor Relapse by Reprogramming Antitumor Immunity. Adv. Healthc. Mater..

[115] Yu S., Li J., Zhang J., Zeng G., Zeng B., Song S., Lao Z., Chen H., Wen Z., Yang Z. (2025). Nanosized shikonin disrupts tumor-cell mismatch repair and synergizes with manganese to sensitize squamous carcinoma to immunotherapy. ACS Nano.

[116] Yi Q., Xia F., Li T.-F., Wang J., Li L.-G., Tan F., Wang X., Tang H., Liu W., Xu Z. (2026). Nano-hydrogel-based delivery system for dihydroartemisinin to enhance cuproptosis and synergize with anti-PD-1 therapy in triple-negative breast cancer. J. Nanobiotechnology.

[117] Lu S., Li Y., Yu Y. (2024). Glutathione-scavenging celastrol-Cu nanoparticles induce self-amplified cuproptosis for augmented cancer immunotherapy. Adv. Mater..

[118] Dalangood S., Hu C., Yuan C., Li X., Qiao W., Li H., Zhang R., Li L., Li P., Yu X. (2025). Cancer-associated adipocytes mediate CD8+ T cell dysfunction via FGF21-driven lipolysis. Cell Rep..

[119] Li Y., Zhang H., Wei J., Jin W., Tong Q., Ye Y., Xie Q., Li Q., Cheng G., Xiong Y. (2025). Celastrol liposomes enhance anti-PD-L1 in triple-negative breast cancer by reprogramming immunosuppressive microenvironment driven by cancer-associated adipocytes. Phytomedicine.

[120] Ji A., Yang P., Ma B., Li J., Yu Y., Zhang X., Chen P., Yao H., Shi L., Hu H. (2025). Photothermal-responsive curcumin-loaded copper-based nanocomposites for targeted drug release and combined immunotherapy. J. Colloid. Interface Sci..

[121] Ge Y.-X., Zhang T.-W., Zhou L., Ding W., Liang H.-F., Hu Z.-C., Chen Q., Dong J., Xue F.-F., Yin X.-F. (2022). Enhancement of anti-PD-1/PD-L1 immunotherapy for osteosarcoma using an intelligent autophagy-controlling metal organic framework. Biomaterials.

[122] Liao Y., Zhao C., Pan Y., Guo Y., Liu L., Wu J., Zhang Y., Rao L., Li Q. (2024). Genetically engineered cellular nanoparticles loaded with curcuminoids for cancer immunotherapy. Theranostics.

[123] Lan J., Zeng R., Li Z., Yang X., Liu L., Chen L., Sun L., Shen Y., Zhang T., Ding Y. (2024). Biomimetic nanomodulators with synergism of photothermal therapy and vessel normalization for boosting potent anticancer immunity. Adv. Mater..

[124] Cheng Y., Han X., Lai X., Wei X. (2024). Liposomal honokiol inhibits non-small cell lung cancer progression and enhances PD-1 blockade via suppressing M2 macrophages polarization. Phytomedicine.

[125] Li X., Zhu S., Yin P., Zhang S., Xu J., Zhang Q., Shi S., Zhang T. (2021). Combination immunotherapy of chlorogenic acid liposomes modified with sialic acid and PD-1 blockers effectively enhances the anti-tumor immune response and therapeutic effects. Drug Deliv..

[126] Dixon S.J., Olzmann J.A. (2024). The cell biology of ferroptosis. Nat. Rev. Mol. Cell Biol..

[127] Agarwala Y., Brauns T.A., Sluder A.E., Poznansky M.C., Gemechu Y. (2024). Targeting metabolic pathways to counter cancer immunotherapy resistance. Trends Immunol..

[128] Liu L., Hao Z., Yang X., Li Y., Wang S., Li L. (2025). Metabolic reprogramming in T cell senescence: A novel strategy for cancer immunotherapy. Cell Death Discov..

[129] Altea-Manzano P., Decker-Farrell A., Janowitz T., Erez A. (2025). Metabolic interplays between the tumour and the host shape the tumour macroenvironment. Nat. Rev. Cancer.

[130] Nunes J.B., Ijsselsteijn M.E., Abdelaal T., Ursem R., van der Ploeg M., Giera M., Everts B., Mahfouz A., Heijs B., de Miranda N.F.C.C. (2024). Integration of mass cytometry and mass spectrometry imaging for spatially resolved single-cell metabolic profiling. Nat. Methods.

[131] Tian R., Xue Z., Chen Y., Qi Y., Zhang J., Yuan J., Ruan D., Lin J., Liu J., Wang D. (2025). Integrating cross-sample and cross-modal data for spatial transcriptomics and metabolomics with SpatialMETA. Nat. Commun..

[132] Desharnais L., Sorin M., Rezanejad M., Liu B., Karimi E., Atallah A., Swaby A.M., Yu M.W., Doré S., Hartner S. (2025). Spatially mapping the tumour immune microenvironments of non-small cell lung cancer. Nat. Commun..

[133] Tong F., Wang Y., Gao H. (2024). Progress and challenges in the translation of cancer nanomedicines. Curr. Opin. Biotechnol..

[134] Maheshwari R., Kapoor D., Polaka S., Bhattacharya S., Prajapati B. (2025). Roadmap for commercial nanomedicine development: Integrating quality by design principles with pharmaceutical nanotechnology. Mol. Pharm..

[135] Cisneros E.P., Morse B.A., Savk A., Malik K., Peppas N.A., Lanier O.L. (2024). The role of patient-specific variables in protein corona formation and therapeutic efficacy in nanomedicine. J. Nanobiotechnology.

[136] Yang H., Wang J., Zhou X., Fu D., Le C., Wei J., Qian Y., Fang Y., Jin T., Kong X. (2024). Impact of protein corona and PEGylation on the accumulation and efficacy of lysosome-disrupting lipid nanoparticles in KRAS-mutated cancer. Nano Today.

[137] Shen X., Pan D., Gong Q., Gu Z., Luo K. (2024). Enhancing drug penetration in solid tumors via nanomedicine: Evaluation models, strategies and perspectives. Bioact. Mater..

