# Supplementary material for: Engineering Natural Product Nanomedicines to Modulate Tumor Immunometabolism for Enhanced Cancer Immunotherapy
Source: Molecules. 2026 Nov 3;31(21):3965. doi: 10.3390/molecules31213965 (PMC13765510; doi:10.3390/molecules31213965)
Supplement: Supplementary file 1 [file molecules-31-03965-s001.zip › molecules-4594551-supplementary.pdf]

Supplementary Materials

Supplementary Table S1. Mechanistic Validation and Supporting Evidence

Study/formulation	Mechanistic validation	Component-specific controls	PK/biodistribution
Emodin-loaded iron-based nanoparticles	Lactate measurement; TAM markers (CD16/32, CD206) and TNF-α	Free emodin; unloaded iron-based nanoparticles	Fluorescence imaging showed tumor accumulation 24 h after intravenous injection; ICP-MS quantified iron in tumors and major organs, with prominent distribution in the spleen and lungs.
Shikonin/JQ1 nanoparticles	Glycolytic changes; DC maturation; $CD8^+$ T-cell infiltration	Free SHK, SHK + JQ1, LF NPs and Man-LF NPs; combination and targeting benefits	Man-LF NPs showed increased tumor accumulation, attributed to mannose receptor- and LRP-1-mediated targeting of tumor cells and TAMs.
DHA-containing nanohydrogel	Copper chelation (TTM, DPA); cuproptosis-related proteins; DLAT oligomerization	DHA-, CuS- and complete-formulation controls	Fluorescence imaging showed sustained tumor retention at day 4, with additional fluorescence detected in the liver. Drug release approached 88% by day 4 and exceeded 90% by day 6 under pH 5.0 plus 10 mM H_2O_2.
Celastrol-Cu nanoparticles	GSH depletion; copper-dependent cell death; ICD-related outcomes	Celastrol-, copper- and nanoparticle-related controls	Cy5.5-labelled Cel-Cu NPs accumulated in subcutaneous 4T1 tumors after intravenous injection; ex vivo imaging showed distribution in the tumor and major organs at 48 h.
Curcumin-Cu_9S_8 nanocomposites	ROS; mitochondrial dysfunction; DAMP release; EGFR-STAT3/PD-L1	Carrier, curcumin, laser and combination controls	Not reported
